# A new genus and species of marine catfishes (Siluriformes; Ariidae) from the upper Eocene Birket Qarun Formation, Wadi El-Hitan, Egypt

**DOI:** 10.1371/journal.pone.0172409

**Published:** 2017-03-01

**Authors:** Sanaa E. El-Sayed, Mahmoud A. Kora, Hesham M. Sallam, Kerin M. Claeson, Erik R. Seiffert, Mohammed S. Antar

**Affiliations:** 1 Mansoura University Vertebrate Paleontology Center (MUVP), Department of Geology, Faculty of Science, Mansoura University, Mansoura, Egypt; 2 Department of Anatomy, Philadelphia College of Osteopathic Medicine, Philadelphia, Pennsylvania, United States of America; 3 Department of Cell and Neurobiology, Keck School of Medicine, University of Southern California, Los Angeles, California, United States of America; 4 Department of Geology and Paleontology, Nature Conservation Sector, Egyptian Environmental Affairs Agency, Cairo, Egypt; University of Michigan, UNITED STATES

## Abstract

Wadi El-Hitan, the UNESCO World Heritage Site, of the Fayum Depression in the northeast part of the Western Desert of Egypt, has produced a remarkable collection of Eocene vertebrates, in particular the fossil whales from which it derives its name. Here we describe a new genus and species of marine catfishes (Siluriformes; Ariidae), *Qarmoutus hitanensis*, from the base of the upper Eocene Birket Qarun Formation, based on a partial neurocranium including the complete left side, partial right dentary, left suspensorium, two opercles, left pectoral girdle and spine, nuchal plates, first and second dorsal spines, Weberian apparatus and a disassociated series of abdominal vertebrae. All of the elements belong to the same individual and some of them were found articulated. *Qarmoutus* gen. nov. is the oldest and the most complete of the Paleogene marine catfishes unearthed from the Birket Qarun Formation. The new genus exhibits distinctive features not seen in other African Paleogene taxa, such as different sculpturing on the opercle and pectoral girdle with respect to that on the neurocranium and nuchal plates, denticulate ornamentation on the skull bones arranged in longitudinal rows and forming a radiating pattern on the sphenotic, pterotic, extrascapular and the parieto-supraoccipital, indentations or pitted ornamentation on the nuchal plates as well as the parieto-supraoccipital process, strut-like radiating pattern of ornamentation on the opercle from the proximal articulation to margins, longitudinal, curved, reticulate ridges and tubercular ornamentations on the cleithrum, sinuous articulation between the parieto-supraoccipital process and the anterior nuchal plate, long, narrow, and arrowhead shaped nuchal shield, very small otic capsules restricted to the prootic. Multiple parsimony and Bayesian morphological phylogenetic analyses of Ariidae, run with and without “molecular scaffolds”, yield contradictory results for the placement of *Qarmoutus*; the genus is either a phylogenetically basal ariid, or it is deeply nested within the ariid clade containing New World species of *Sciades*.

## Introduction

The Valley of Whales or Wadi El-Hitan has been a UNESCO World Heritage Site since 2005. The valley is located northwest of the Fayum Depression, north of the Western Desert of Egypt, and preserves the richest marine mammal-bearing Paleogene exposures in Egypt, if not the entire Afro-Arabian landmass, with a remarkable collection of Eocene fossil whales from which it derives its name [1,2,3]. The fossil whales from Wadi El-Hitan have been famous for their completeness and exquisite preservation, with the majority of them being assigned to the late Eocene taxa *Basilosaurus isis* and *Dorudon atrox* [2,4]. In addition to the whale fossils, Wadi El-Hitan has produced a wide variety of other vertebrate fauna such as crocodiles [5], sirenians [6,7] and fishes [8,9]. However, catfish fossils had not previously been documented from the Paleogene deposits of Wadi El-Hitan, despite decades of intensive paleontological sampling.

Catfishes or Siluriformes represent ~ 22% of all freshwater fishes and have had a widespread distribution on all continents [10]. The marine Siluriformes are represented by two major clades—Plotosidae and Ariidae [11]. The catfishes of the family Ariidae have a wide geographic distribution, being found in the tropical and subtropical continental shelves of the Atlantic, Indian, and Pacific oceans. However, some members of this family inhabit brackish estuaries and some are found only in either freshwater or marine environments [12]. Ariidae is a clade of catfishes that has been supported by several studies [13,14,15,16]. Based on molecular phylogenetic analyses, Betancur-R et al. [17] recognized two major subfamilies for the family Ariidae—Ariinae and Galeichthyinae. The morphological studies of Marceniuk et al. [18] suggest the existence of a new subfamily, Bagreinae, increasing the number of ariid subfamilies to three.

The Paleogene fossil record of the catfishes in Africa documents six major families: Ariidae, Bagridae, Clariidae, Claroteidae, Mochokidae and Schilbidae. The ariid catfish fossil record in Africa (with the exception of the Egyptian fossils) is based on fragmentary spines and otoliths [19] from the middle Eocene of Nigeria; however, this record may be erroneous [20]. The first study of Fayum catfishes was undertaken by Stromer [21], who named and described two genera from younger deposits of the upper Eocene Qasr el-Sagha Formation: *Fajumia schweinfurthi* and *Socnopaea grandis*; he did not place either into any known family. Subsequently, Peyer [22] restudied more fossil material of *Fajumia schweinfurthi* and *Socnopaea grandis* and described two new species from the Qasr el-Sagha Formation—one of which was placed in *Fajumia* (*Fajumia stromeri*), and one which was placed in a new genus and species as *"Ariopsis aegyptiacus*", later renamed *Eopeyeria aegyptiaca* by Whitely [23], (see also Ferraris [24]). Peyer assigned *Eopeyeria aegyptiaca* and, a new species of a marine catfish from the middle Eocene Mokattam Formation near Cairo, *Arius fraasi*, to the family Ariidae. After half a century, Greenwood [25] suggested that the Paleogene catfishes discovered from Egypt by Stromer and Peyer are members of the Ariidae family and listed them as a part of his revision of Cenozoic freshwater fish faunas from Africa.

Cranial and postcranial siluriform elements have also been reported from almost all quarries of upper Eocene-lower Oligocene age (~34–29 Ma) in the Jebel Qatrani Formation exposed in the northern part of the Fayum Depression; however none of those elements were assigned to the genus level [26]. The only catfish materials described thus far from the Birket Qarun Formation were unearthed from the locality BQ-2, and are based on isolated elements that belong to the freshwater families Claroteidae and Mochokidae, but none of which was complete enough to be assigned to the genus level [27]. Claroteidae and Mochokidae have also been reported from the “middle” Eocene of Libya based on a skull fragment and tooth, respectively [28]. Here we describe, for the first time, a new ariid catfish genus and species from the marine deposits of the upper Eocene Birket Qarun Formation exposed in the Wadi El-Hitan site of the Fayum Depression of northern Egypt. We also have investigated the phylogenetic position of the new material by adding it to the morphological character matrix of Marceniuk et al. [18].

## Geological setting

The geology of the Wadi El-Hitan area is uncomplicated, consisting of a series of escarpments of middle and late Eocene, early Oligocene, and Miocene age, which are represented by five formations from bottom to top: Gehannam Formation, Birket Qarun Formation, Qasr el-Sagha Formation, Jebel Qatrani Formation and Khashab Formation. The Birket Qarun Formation, which is the main focus of this work, makes up the majority of the base, which includes numerous isolated hills in the Wadi El-Hitan area. Recent field studies on one of these hills have led to the discovery of a well-preserved skull fragment of a large siluriform catfish.

**Fig 1:**
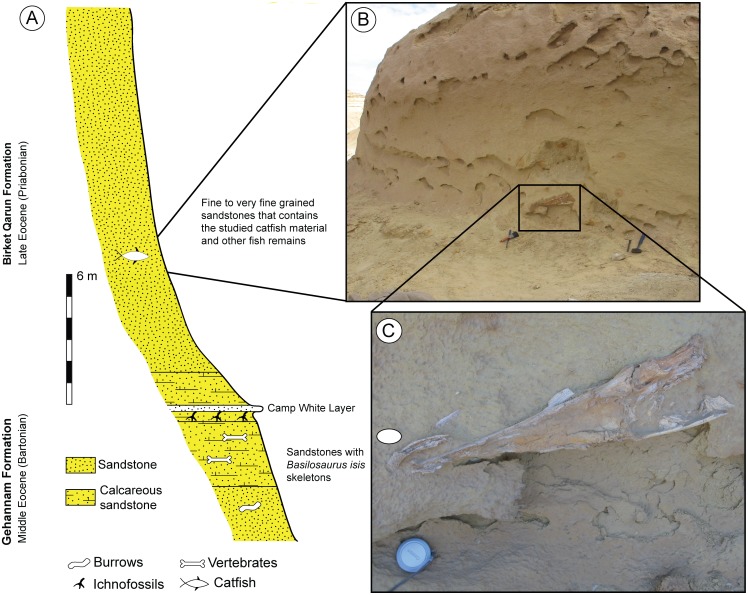
The fossil-bearing interval at Wadi El-Hitan. **A,** Location of the Birket Qarun outcrop in the Fayum Depression; **B,** Measured stratigraphic section at the catfish hill showing the location of *Qarmoutus hitanensis*; **C**, New catfish materials exposed in the fine sandstone of the Birket Qarun Formation; **D,** Close-up view of *Qarmoutus hitanensis* left neurocranium in situ.

A lithologic section at the catfish site (Fig 1) was measured in order to locate the stratigraphic level of the catfish fossil, which was unearthed from the lower level of the Birket Qarun Formation. At Wadi El-Hitan, this unit consists primarily of fine to very fine grained sandstones and greyish black shale beds [29,30]. It overlies conformably the Gehannam Formation, from which it is separated by a well-recognized and widespread marker bed called the Camp White Layer [31]. The new catfish material was collected from ~7 m above the Camp White Layer in the sandstone unit that forms the majority of the base of the Birket Qarun Formation (Fig 1A). Whales and numerous shark taxa were also collected from the bottom of the Birket Qarun Formation [2,8]

Different paleontological studies on the Birket Qarun Formation of Wadi El-Hitan date the formation as late Eocene (Priabonian) (e.g., [30,32,33]). The depositional environment of the Birket Qarun Formation has long been a matter of debate. A shallow marine shelf in offshore barrier bar settings was suggested by Gingerich [31] and Anan and El Shahat [34]. Others suggested less restricted conditions for the Formation based on the presence of intense bioturbation [35].

## Materials and methods

The new fossil catfish (MUVP 58) is remarkably well-preserved and consists of an incomplete neurocranium that preserves all of the left side, partial right dentary, left suspensorium, two opercles, left pectoral girdle and spine, nuchal plates, first and second dorsal spines, Weberian apparatus and a disassociated series of vertebrae belonging to the same individual. All the catfish elements were collected in one medium jacket and some of the skull bones were found articulated in their natural position.

The specimen was collected during a student training field exploration effort of the Mansoura University Vertebrate Paleontology Center (MUVP) in collaboration with the Egyptian Environmental Affairs Agency (EEAA), based on the mutual memorandum of understanding between the two institutions. MUVP 58 is housed at the Mansoura University Vertebrate Paleontology Center, Department of Geology, Mansoura University and underwent preparation at its facilities. MUVP 58 elements were originally entombed in a fine sandstone matrix, which became fully embedded in sutures, foramina and cracks. Preparing of the entire matrix is not only a difficult task, but also weakens the specimens and makes them vulnerable to breakage. The specimen was mechanically prepared under a microscope and was photographed with a thin coat of Ammonium Chloride (NH4Cl) to whiten the specimens for the figures presented here. The terminology of the anatomical features mentioned in the text is modified after Longbottom [36]. The terminology and measurements of the pectoral fin spines are following Vanscoy et al. [37].

We ran multiple phylogenetic analyses to determine the placement of *Qarmoutus* within Ariidae, employing an updated version of the 230-character, 93-taxon morphological character matrix published by Marceniuk et al. [18]. Following those authors, 35 of the multistate characters in the matrix were treated as ordered (supporting dataset 1). *Qarmoutus* could be scored for 87 characters. Using both parsimony and Bayesian methods, we first analyzed the matrix with no topological constraints, and then with relationships among extant species constrained to be consistent with the best-supported nodes in the most comprehensive molecular phylogenetic analysis of Ariidae [38]; i.e., Bayesian posterior probabilities >0.95 in Fig 1 of that paper]. In the constrained analyses, *Qarmoutus* and extant species not sampled for molecular data by Betancur-R [38] were free to fall anywhere in tree. Parsimony analyses were run for 10,000 replicates in PAUP 4.0b10 [39], with TBR branch swapping and random addition sequence; bootstrap support is based on 1,000 pseudoreplicates. Bayesian analyses were run in MrBayes 3.2.2 [40] for 10 million MCMC generations, with three cold chains and one hot chain (temp = 0.02); chains were sampled every 1,000 generations and these results are summarized using an “allcompat” (majority-rule consensus with compatible groups) tree with posterior probabilities for each clade in that consensus tree.

No permits were required for the described study, which complied with all relevant regulations. 3D digital materials are also available for viewing and direct download at http://morphosource.org/Detail/SpecimenDetail/Show/specimen_id/4462

### Nomenclatural acts

The electronic edition of this article conforms to the requirements of the amended International Code of Zoological Nomenclature, and hence the new names contained herein are available under that Code from the electronic edition of this article. This published work and the nomenclatural acts it contains have been registered in ZooBank, the online registration system for the ICZN. The ZooBank LSIDs (Life Science Identifiers) can be resolved and the associated information viewed through any standard web browser by appending the LSID to the prefix "http://zoobank.org/". The LSID for this publication is: urn:lsid:zoobank.org:pub: 133E807A-C70F-4B1B-BEBF-5B71FCB98AF2. The electronic edition of this work was published in a journal with an ISSN, and has been archived and is available from the following digital repositories: PubMed Central, LOCKSS.

Anatomical abbreviations used in the text are: **ac**, aortic canal; **a.d.pr.cl**, anterior dorsal process of cleithrum; **afo**, anterior cranial fontanelle; **af.psp**, **anp**, anterior nuchal plate; articulatory facet for pectoral spine; **ant.ar,** anterior articulation; **ant.pr,** anterior process; **ap**, metapterygoid anterior process; **ar.co.neuro**, articulating condyle for the neurocranium; **ar.d.pr.cl**, articulation facet with the dorsal process of cleithrum; **ar.fa. ang.art**, articulating facet for angulo-articular; **ar.fa.hyo**, articulation facet for hyomandibula; **ar.pstt,** articulation facet for posttempro-supracleithrum; **arr.d.dd,** area for attachment of the dorsal division of arrector dorsalis muscle; **arr.d.vd**, area for attachment of the ventral division of the arrector dorsalis muscle; **art.f,** articular foramen; **art.pnp**, articulation with the posteriopr nuchal plate; **ba**, basioccipital; **cl**, cleithrum; **clap**, crest for insertion of the levator arcus palatini muscle; **cl.hp**, humeral process of cleithrum; **dctr**, dorsal crest of tripus; **d.ml**, dorso-medial limp of posttempro-supracleithrum; **do.pr**, dorsal process for insertion of the dilatator operculi muscle; **d.pr.cl**, dorsal process of cleithrum; **exo**, exioccipital; **ext**, extrascapular; **fc**, facial canal; **fgn**, foramen for the glossopharyngeal nerve; **fm,** mandibularis foramen; **fn**, fenestra; **fopth**, foramen for ophthalmic nerve; **fr**, frontal; **ftr**, foramen for trigeminofacial nerve; **fvn**, foramen for vagus nerve; **hyo**, hyomandibula; **hyo.bl**, hyomandibular blade; **hyo.pr**, hyomandibular process; **le**, lateral ethmoid; **le.lh**, lateral ethmoid lateral horn; **lnp**, lateral nuchal plate; **l.pr**, lateral process; **me**, mesethmoid; **mg**, medial groove of the neurocranium; **mto**, metapterygoid; **oac**, opening of arotic canal; **of**, optic foramen; **opf,** opercle facet for hyomandibular; **orb**, orbitosphenoid; **pah**, adductor hyomandibularis process for hyomandibular; **pas**, parasphenoid; **p.d.pr.cl**, posterior dorsal process of cleithrum; **pnp,** posterior nuchal plate; **pop**, preopercle; **pr**, prootic; **prp4**, parapophysis of the fourth vertebra; **prp5**, parapophysis of the fifth vertebra;; **psp,** pectoral spine**; psp.ac**, pectoral spine anterior condyle; **psp.dc**, pectoral spine dorsal condyle; **psp.gr**, pectoral spine groove; **psp.vc,** pectoral spine ventral condyle; **pt**, pterotic; **pts**, pterosphenoid; **pt.sp**, pterotic spine; **q**, quadrate; **r.lop,** ridge for insertion of the levator operculi muscle; **sc**, symplectic canal; **scor**, scapulo-coracoid; **scor.br**, scapulo-coracoid bridge; **scor.pl.fr**, scapulo-coracoid postero-lateral foramen; **scor.pl.pr**, scapulo-coracoid postero-lateral process; **scor.r**, scapulo-coracoid ridge; **scp**, sensory canal pore; **sp**, sphenotic; **suoc**, parieto-supraoccipital; **suoc. pr**, parieto-supraoccipital process; **svp**, subvertebral process; **tptr**, transformator process of the tripus; **tr**, tripus; **tr.ind**, triangular indentation; **v.ll**, ventro-lateral limp of posttempro-supracleithrum; **v.ml**, ventro-medial limp of posttempro-supracleithrum; **vo**, vomer; **vtp**, vomerine tooth plate; **v1**, first vertebra; **v5**, fifth vertebra; **W.cc**, Weberian compound cenrum; **1**^**st**^ **ptg**, first pterygiophore; **2**^**nd**^ **ptg**, second pterygiophore; **3**^**rd**^ **ptg**, third pterygiophore.

## Systematic paleontology

Order: **Siluriformes** Sensu Fink and Fink, 1996 [41]

Family: **Ariidae** Bleeker, 1862 [42]

***Qarmoutus***, gen. nov.

ZooBank Life Science Identifier (LSID) for the genus: zoobank.org:act:8FCC2E83-D5FE-47F1-83BD-60B5AD320E8E

**Type species**. *Qarmoutus hitanensis*, new species

**Etymology.** *Qarmout* Arabic word for catfish–gender masculine

**Generic diagnosis.** As for the type species.

**Type locality.** The UNESCO World Heritage Site, Wadi El-Hitan, Fayum Depression, Western Desert, Egypt.

***Qarmoutus hitanensis*** sp. nov.

(Figs 2–17, Table 1)

**Table 1:** Measurements of *Qarmoutus hitanensis* gen. et sp. nov following Longbottom [36].

Specimen	fr-pt length mm	fr-ext length mm	pt-suoc width mm	fr-pt length divided by pto-suoc width	fr-ext length divided by pt/suoc width
*Qarmoutus hitanensis* (gen. et sp. nov).	63	80	70	0.9	1.14

fr—pt is the distance from the sphenotic—frontal—parieto-supraoccipital junction to the pterotic—extrascapular—parieto-supraoccipital junction, fr—ext is the distance from the sphenotic—frontal—parieto-supraoccipital junction to the extrascapular posterior border, pt—suoc is the width of the parieto-supraoccipital at the sphenotic -pterotic- parieto-supraoccipital junctions.

ZooBank LSID for the species: urn:lsid:zoobank.org:act:4F9F163B-53C5-40A4-80FA-84BC2B8DB0D7

**Etymology.** *hitanensis* in reference to the UNESCO World Heritage Site, Wadi El-Hitan, Fayum Depression, Western Desert, Egypt.

**Holotype.** MUVP 58; associated elements of a single individual composed of a nearly complete neurocranium, partial right dentary, paired opercles, left suspensorium, left pectoral girdle (cleithrum articulated with pectoral spine), first and second dorsal spines, nuchal plates, Weberian apparatus and three disarticulated abdominal vertebrae.

**Type locality and age.** The MUVP 58 specimen was excavated from the upper Eocene (Priabonian ~37 Ma) deposits of the Birket Qarun Formation in Wadi El-Hitan, Fayum Depression, northern Western Desert, Egypt.

### Diagnosis

*Qarmoutus hitanensis* is distinguished from the remaining ariid genera by the following unique characters (1–8) and characters that are shared with various extant taxa (9–16): (1) different sculpturing on the opercle and pectoral girdle with respect to that on the neurocranium and nuchal plates; (2) denticulate ornamentation on the skull bones arranged in longitudinal rows and forming a radiating pattern on the sphenotic, pterotic, extrascapular and the parieto-supraoccipital; (3) indentations or pitted ornamentation on the nuchal plates as well as the parieto-supraoccipital process; (4) strut-like radiating pattern of ornamentation on the opercle from the proximal articulation to margins; (5) longitudinal, curved, reticulate ridges and tubercular ornamentations on the cleithrum; (6) sinuous articulation between the parieto-supraoccipital process and the anterior nuchal plate; (7) long, narrow, and arrowhead shaped nuchal shield; (8) very small otic capsules restricted to the prootic; (9) large size (shared with *Eopeyeria aegyptiaca*); (10) distinct nuchal plates (shared with *Galeichthys*, *Bagre bagre* and *Eopeyeria aegyptiaca*); (11) absence of the posterior cranial fontanel (shared with *Batrachocephalus* and *Sciades*); (12) indistinct epiphyseal bar (shared with *Batrachocephalus* and *Sciades*); (13) weakly differentiated otic capsules (shared with *Cathorops*); (14) absence of basioccipital lateral process (shared with *Galeichthys* and *Eopeyeria aegyptiaca*); (15) absence of metapterygoid anterior process (shared with *Batrachocephalus*, *Ketengus* and *Osteogeneiosus*) and (16) very long humeral process of the cleithrum (shared with *Cinetodus* and *Pachyula*).

### Description

MUVP 58 is an extraordinarily preserved and semi-articulated specimen. The specimen was not subjected to major post-depositional forces, and, thus, structural deformation. However, the specimen bears several cracks that occurred during collection and transportation. The collected materials comprise several bony elements, all of which belong to the same individual. Moreover, a number of small fish vertebrae, shark and ray teeth and bivalves are preserved as associated fauna.

#### Skull roof elements

**Fig 2:**
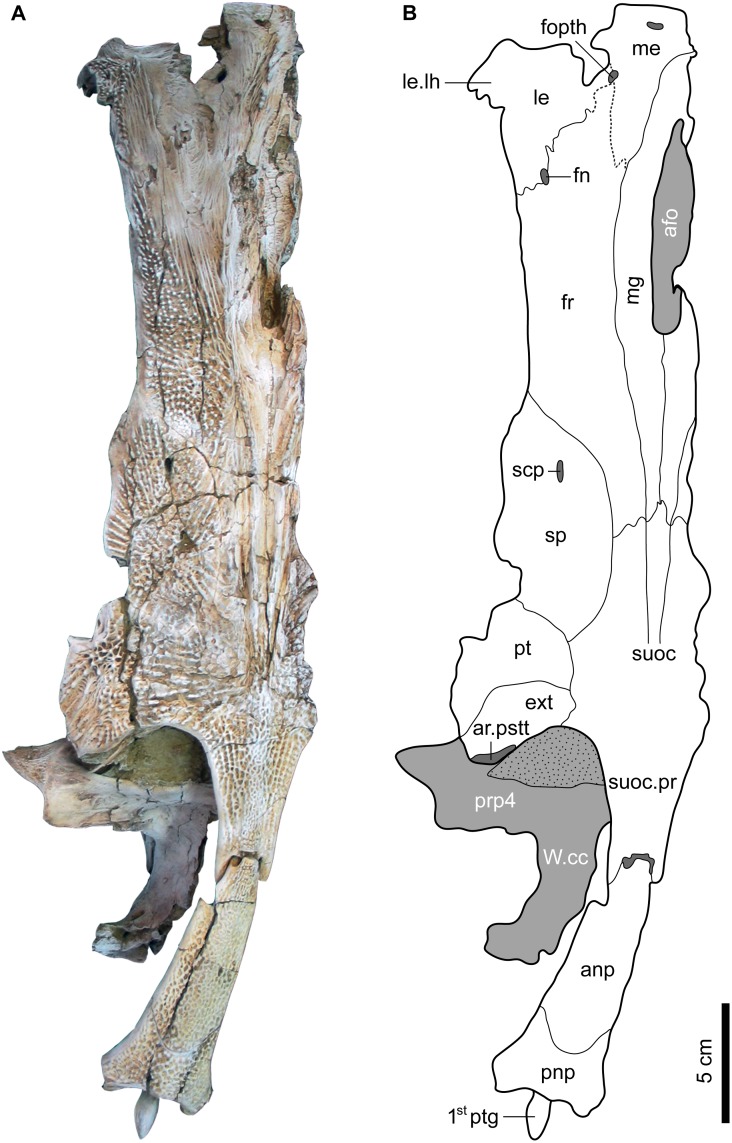
Dorsal view of the left neurocranium and nuchal plates of *Qarmoutus hitanensis* gen. et sp. nov. **A,** Photograph and **B,** Line drawing showing the anatomical features mentioned in the text.

**Fig 3:**
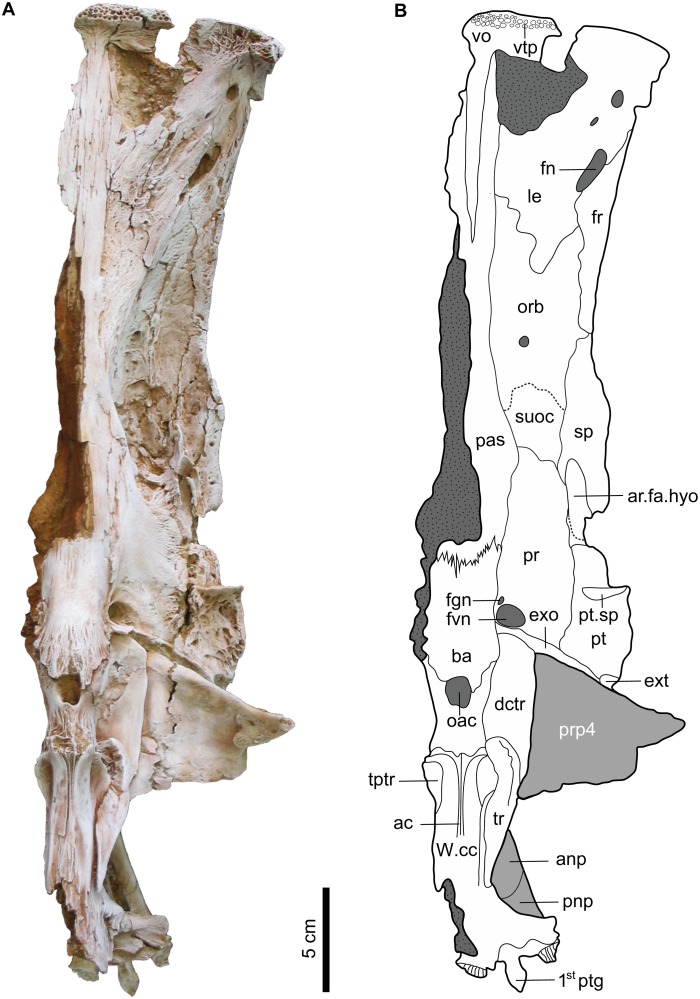
Ventral view of the left neurocranium and nuchal plates of *Qarmoutus hitanensis* gen. et sp. nov. **A,** Photograph and **B,** Line drawing showing the anatomical features mentioned in the text.

The skull roof bones of *Qarmoutus hitanensis* (Figs 2 and 3) are flat, giving the skull a somewhat rectangular shape, which is broad anteriorly and posteriorly, with the narrowest point being at about the midpoint. The suture lines between the cranial elements are preserved, however in some cases it is difficult to confidently trace them. The anterior two-thirds of the neurocranium are dorso-ventrally compressed. On the dorsal surface of the neurocranium, the ornamentation differs among the skull roof elements. There are longitudinal rows of slightly raised ridges and tubercles on the lateral ethmoid and the frontal, whereas a radiating pattern ornaments the sphenotic, pterotic, extrascapular and the parieto-supraoccipitl. The tubercles (denticles) are well-developed and semi-spherical, 2–3mm high with blunt tips and are arranged in lines and radiate out from the center of the parieto-supraoccipital and sphenotic.

Mesethmoid (me). This median unpaired bone at the anterior most part of the neurocranium is only partially preserved in MUVP 58, which makes it difficult to determine the degree of the development of the anterolateral cornua. The mesethmoid is broad anteriorly and its posterior branch is long, broad and delimiting one quarter of the length of the anterior cranial fontanelle (afo). In dorsal view, the mesethmoid meets the lateral ethmoid (le) and the frontal (fr) laterally and the medial groove of the neurocranium (mg) medially, but the suture lines between the two bones are not clear and represented by dashed lines in (Fig 2). A small fenestra delimited by the mesethmoid and the lateral ethmoid is present dorsally. Ventrally, the mesethmoid region is damaged, which makes it difficult to determine the boundary between the mesethmoid and the adjacent bones (Fig 3). The bone is slightly ornamented with ridges on its dorsal surface (Fig 2).

Lateral ethmoid (le). A large flat paired bone with a roughly triangular shape occupies the antero-lateral part of the neurocranium and is situated lateral to the mesethmoid and anterior to the frontal (Fig 2). The latter widely overlap the lateral ethmoid dorsally. The lateral ethmoid contacts the frontal in a curved zig-zagged suture that delimits a small oval fenestra. In lateral view, the lateral horn of lateral ethmoid is evident, slightly compressed and acute, short and laterally oriented and is slightly curved. There is a distinct deep circular pit for the superficial ophthalmic nerve (fopth) between the lateral ethmoid and the mesethmoid. In ventral view, it connects to the parasphenoid (pas) medially, the orbitosphenoid (orb) through a zig-zagged suture posteriorly and the frontal laterally (Fig 3). There are three foramina of different sizes on the ventral margin of the lateral ethmoid. The dorsal surface of the bone is ornamented by tubercles and longitudinal ridges that are antero-posteriorly directed (Fig 2).

Frontal (fr). The frontal bone is flat, antero-posteriorly elongate and broader anteriorly than posteriorly. Only the left frontal is preserved and separated from the right frontal by the mesethmoid and the medial groove of the neurocranium (mg). It articulates antero-laterally with the lateral ethmoid via a zig-zag suture (Fig 2), posteriorly with the parieto- supraoccipital, and postero-laterally with the sphenotics (sp) in a smooth concave suture. Medially, the frontal has a laminar projection between the mesethmoid and the lateral ethmoid. The frontal surface has no evident foramina. Ventro-medially, it articulates with the lateral ethmoid and the orbitosphenoid (Fig 3). The anterior cranial fontanelle (afo) is present anterior to the parieto-supraoccipital (suoc) between the frontals and the mesethmoid bones (Fig 2). It is roughly oval-shaped, with a narrow portion that projects anteriorly between the posterior branches of the mesethmoid. The posterior cranial fontanelle is absent. The medial groove of the neurocranium (mg) is shallow and runs antero-posteriorly, terminating anteriorly in the mesethmoid and ends posteriorly continuing onto about the anterior third of the parieto-supraoccipital. The walls of the medial groove of the neurocranium are ornamented with a single row of long tubercles. The dorsal surface of the bone is highly ornamented with tubercles.

Parieto-supraoccipital (suoc). The parietal is fused with supraoccipital forming the parieto-supraoccipital bone, a synapomorphy of Siluriformes [43]. The parieto-supraoccipital is one of the largest bones of the dorsal skull roof, and forms the majority of the posterior-most dorsal region of the neurocranium (Fig 2). The posterior portion of the parieto-supraoccipital is narrower when compared to its anterior portion with the maximum width at the midpoint of the bone, contributing to a somewhat diamond-shape appearance. The anterior-most edge of the parieto-supraoccipital is sutured with the left and right frontals. The suture line between the parieto-supraoccipital and the frontals is clear and well-preserved. The anterior most part of the parieto-supraoccipital projects slightly between the frontal bones anteriorly in the midline (Fig 2). The medial groove of the neurocranium extends posteriorly into the first 1/3 of the parieto-supraoccipital bone. In dorsal view, the parieto-supraoccipital is sutured with the sphenotic, pterotic (pt) and extrascapular (ext) laterally with no fenestra between them and extends posteriorly via the parieto-supraoccipital process (suoc.pr) to articulate with the anterior nuchal plate (anp). The articulation between the parieto-supraoccipital and the anterior nuchal plate is broad, resulting from the wide spacing between two bones in dorsal view (Fig 2). The parieto-supraoccipital is slightly narrower anteriorly at the frontal-sphenotic suture than at the sphenotic-pterotic suture and the pterotic-extrascapular suture and meets with the sphenotic at its antero-lateral corner. In ventral view, a small portion of the parieto-supraoccipital appears and is bounded by the orbitosphenoid anteriorly, the prootic (pr) posteriorly, the parasphenoid medially and the sphenotic laterally. The posterior connections to the parieto-supraoccipital are difficult to determine due to the connection of the Weberian compound centrum (W.cc) to the neurocranium. The parieto-supraoccipital is ornamented with radiating pattern of tubercles that radiate from the center of the bone around the posterior end of the medial groove of the neurocranium. The parieto-supraoccipital process (suoc.pr) is long and narrow and slightly elevated above the level of the skull roof bones (Fig 2). Its posterior margin contacts the anterior nuchal plate (anp) through a curved but sinous articular surface. The ventral crest of the parieto-supraoccipital process is well-developed through the entire extension of the process. The pits of the ornamentation are elongated and shaped like tear drops, differing from the other dorsal skull roof bones.

**Fig 4:**
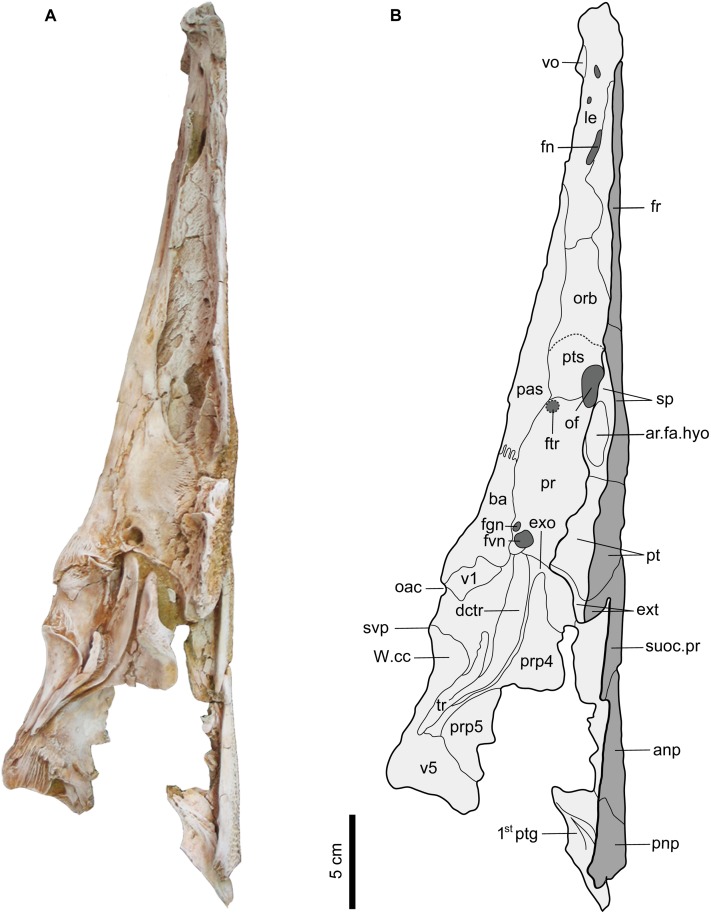
Lateral view of the left neurocranium and nuchal plates of *Qarmoutus hitanensis* gen. et sp. nov. **A,** Photograph and **B,** Line drawing showing the anatomical features mentioned in the text.

Sphenotic (sp). The medium sized and oval-shaped sphenotic is situated roughly on the medio-lateral part of the neurocranium, just before the expansion of the posterior part of the skull roof (Figs 2 and 3). In dorsal view, the bone is antero-posteriorly elongate and sutures antero-medially with the frontal, postero-medially with the parieto-supraoccipital, and posteriorly with the pterotic. There is an oval-shaped and small sensory canal pore (scp) situated at the anterior portion of the sphenotic. In ventral view, the sphenotic articulates with a small portion of the frontal anteriorly, the orbitosphenoid, ventral part of the parieto-supraoccipital and the prootic medially and the pterotic posteriorly. The facet for the hyomandibular articulation (ar.fa.hyo) is large and oval-shaped, occupies the postero-medial corner of the sphenotic, and is independent from the prootic and pterotic (Figs 3 and 4). The bone lacks the sphenotic spine and is covered with radiating pattern of tubercles dorsally.

Pterotic (pt).—The pterotic is posterior to the sphenotic and smaller than it (Fig 2) and forms the anterior lateral expansion of the posterior part of the skull roof. Dorsally, it articulates with the parieto-supraoccipital medially and with the extrascapular posteriorly. The lateral border of the pterotic is wider than the medial border giving it a fan shape. In ventral view, the pterotic sutures with the sphenotic anteriorly, the prootic medially and with the exoccipital (exo) and extrascapular posteriorly. The pterotic spine (pt.sp) is roughly situated in the midway of the bone, somewhat rectangular-shaped and present ventrally on the postero-lateral corner of the bone. The pterotic doesn't contacts the hyomandibula. The dorsal surface of the pterotic is ornamented with tubercles that are connected with each other via low ridges, leading to a net shape. This configuration is similar to that of the dorsal part of the frontal.

Extrascapular (ext). The extrascapular is a laminar bone that forms the postero-lateral corner of the posterior expansion of the neurocranium (Fig 2). The extrascapular has an oval shape and is smaller than the pterotic. In dorsal view, the extrascapular sutures medially with the parieto-supraoccipital and anteriorly with the pterotic. Postero-laterally, there is a groove for the articulation with the posttemporo-supracleithrum (ar.pstt). In ventral view, only a small part of the extrascapular is visible as it is covered largely by the parapophysis of the fourth centrum (prp4) of the Weberian apparatus. The ornamentation configuration of the extrascapular is composed of tubercles connected via low ridges that are arranged in rows.

#### Ventral and lateral elements of the neurocranium

The left ventral neurocranium is complete and bears all of the bones starting from the anterior edge of the vomer (vo) anteriorly to the posterior edge of the basioccipital (ba) posteriorly.

Vomer (vo). The vomer is a thin T-shaped bone that joins the mesethmoid dorsally and the parasphenoid postero-laterally (Fig 3). Posteriorly, the vomer has an elongated needle-like posterior process that extends backward, terminates between the two anterior processes of the parasphenoid and reaches the level of the anterior part of the orbitosphenoid without connecting to it. Laterally, the vomer has a long and wide lateral process. Ventrally, a single large vomerine tooth plate (vtp) is preserved and strongly attached to the vomer and contains four rows of circular tooth pedestals that vary in size. The antero-lateral part of the vomer is damaged during the collecting field process, which makes it difficult to confidently determine the relationship between the vomer and the adjacent bones.

Orbitosphenoid (orb). The bone occupies the middle lateral area of the neurocranium between the left and right orbits. The orbitosphenoid is large and has a butterfly shape in ventral view, with a high vertical wall medially. There is a small and circular foramen at the midpoint of the base of the vertical wall of the orbitosphenoid. The bone is bounded anteriorly by the lateral ethmoid via a zig-zagged suture and laterally with the frontal and sphenotic. The posterior margin of the bone is not clear, so the suture between the orbitosphenoid and the ventral part of the parieto-supraoccipital bone is represented by a dashed line in Fig 3. In lateral view, the anterior medial border of the orbitosphenoid joins the parasphenoid and the posterior medial portion is delimited by the parasphenoid and pterosphenoid.

Pterosphenoid (pts). The pterosphenoid is a small and oval shaped vertical plate that can only be seen from the lateral view (Fig 4). The bone contacts the parieto-supraoccipital above the optic foramen (of), the parasphenoid ventrally, the frontal dorsally and the prootic posteriorly. The pterosphenoid forms the anterior and most of the ventral borders of the optic foramen. The suture between the pterosphenoid and the prootic is present at the posterior part of the ventral border of the optic foramen, excluding the parasphenoid from participating in this foramen. The pterosphenoid contacts the orbitosphenoid anteriorly through an obscured suture that is represented by a dashed line (Fig 4), and situated anterior to the large oval shallow depression of the pterosphenoid, which is present antero-ventral to the optic foramen.

Parasphenoid (pas). The bone is long and narrow with a fork shape and occupies most of the base of the neurocranium. It bifurcates anteriorly due to the accommodation of the posterior process of the vomer and separates the lateral ethmoids. The right half of the parasphenoid is not preserved. The parasphenoid articulates with the vomer anteriorly and lateral ethmoid, orbitosphenoid, parieto-supraoccipital and prootic laterally via smooth sutures that lack any interdigitation. The posterior margin of the parasphenoid with the basioccipital is characterized by a deep interdigitating suture. The lateral wing of the parasphenoid is represented by a moderately developed swelling that occurs at the posterior portion of the bone. The parasphenoid is excluded from the trigeminofacial foramen by the prootic and pterosphenoid.

Prootic (pr). The prootic is a large and slightly bulged bone in the postero-ventral part of the neurocranium (Figs 3 and 4). The bone is elongate antero-posteriorly, giving it an oval shape. The prootic sutures with the ventral part of the parieto-supraoccipital anteriorly, the sphenotic and pterotic laterally, the parasphenoid and basioccipital medially and the exoccipital (exo) posteriorly. The suture between the prootic and the pterotic is wide due to post-mortem displacement. Postero-medially, the prootic bears two nerve foramina; the large foramen is for the vagus nerve (fvn) and the small foramen is for the glossopharyngeal nerve (fgn). The anterior margin of the prootic forms part of the posterior margin of the optic foramen.

Basioccipital (ba). The basioccipital is the posterior-most element of the neurocranial floor. The bone is cylindrical in shape and lacks any lateral expansion. In the most posterior part of the bone, the bony texture is fibrous, with shallow fossae present laterally. The basioccipital joins the parasphenoid anteriorly through a strong interdigitating suture (Fig 3) and articulates with the first vertebra (v1) posteriorly via a smooth suture that could be noticed clearly from the lateral view (Fig 4). Antero-laterally, the basioccipital is sutured with the prootic and with tripus (tr) and exoccipital postero-laterally through smooth sutures. The subvertebral process (svp) is short and wide and well-developed. Its ventral tip is split and its anterior margin is smooth. The nature of contribution of the basioccipital in the foramen magnum is hidden due to the attachment of the Weberian compound centrum with the neurocranium.

Exocciptal (exo). The exoccipital forms part of the postero-lateral corner of the neurocranium. A small part of the exoccipital is visible in ventral view, covered by the parapophysis of the fourth centrum of the Weberian apparatus and the tripus (Fig 3). The exoccipital’s anterior margin contacts the extrascapular and the prootic anteriorly and the basioccipital medially. The ventral-median border of the exoccipital contacts the foramen for the vagus nerve.

Epioccipital. The epioccipital is a bone that also forms part of the postero-lateral corner of the neurocranium. It is completely obscured in MUVP 58 due to the attachment of the Weberian compound centrum posteriorly with the basioccipital. The epioccipital is expected to be present entirely inside the posterior part of the neurocranium as it is not shown in ventral or dorsal views.

#### Dentary

**Fig 5:**
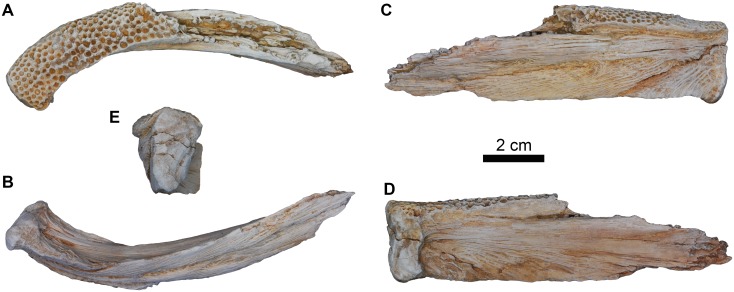
Right dentary fragment of *Qarmoutus hitanensis* gen. et sp. nov. **A,** Dorsal; **B,** Ventral; **C,** Lateral; **D,** Medial and **E,** Symphyseal views.

The dentary is the largest bone of the lower jaw. The anterior part of the right dentary preserves the symphyseal region, and becomes slightly broader anteriorly at the symphyseal joint; it has a nearly 90 degree intermandibular angle (Fig 5). The symphyseal surface is relatively flat with a low symphyseal process that projects postero-ventrally. It has three shallow grooves extending radially from the dorso-medial aspect. The dental lamina bears small numerous tooth pedestals that are present on the entire preserved part of the occlusal surface and arranged in six to seven rows.

In ventral view, the dentary has a long, sharp and very conspicuous antero-ventral crest that tapers anteriorly and bifurcates into two small crests near the symphysis. The antero-ventral crests join the medial and anterior margins of the symphyseal surface, forming a triangular depression. The lateral surface of the dentary possesses two mandibular foramina and has a pronounced surface texture. The latter is formed by imbricated V-shaped ridges, the apices of which occur at the mandibular foramina. The dentary is grooved medially to accommodate the Meckel's cartilage. In medial view, there is a bone shelf projecting from the medial margin of the dentary to support the tooth pedestals. The medial surface has very shallow longitudinal ridges, radiating from the midpoint of the symphysis.

#### Suspensorium

The left suspensorium of MUVP 58 was found separated from, but very close to, the neurocranium. Despite the delicateness of the bone, all of the elements (preopercle, hyomandibula, metapterygoid and quadrate) of the suspensorium are well-preserved aside from some very minor damage.

**Fig 6:**
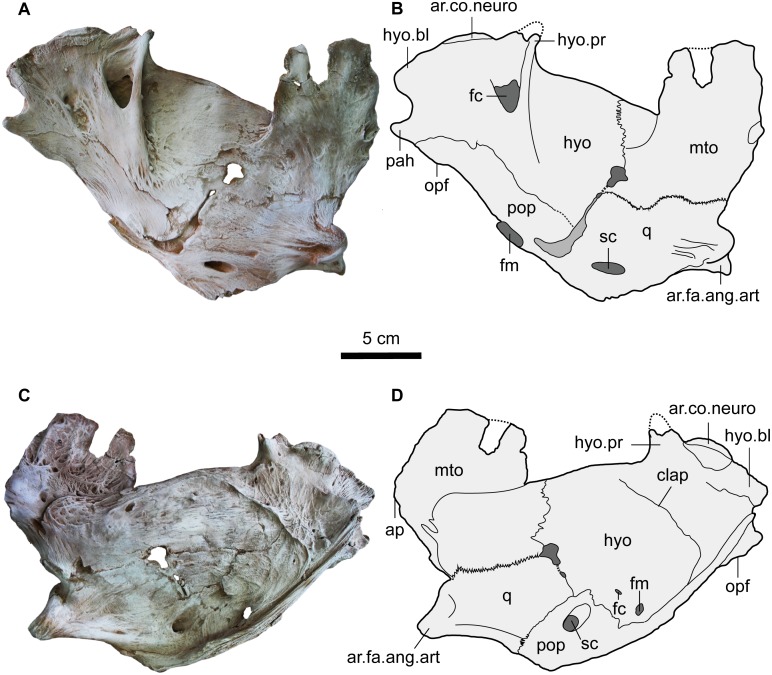
Left suspensorium of *Qarmoutus hitanensis* gen. et sp. nov. Medial view in **A,** Photograph and **B,** Line drawing showing the anatomical features mentioned in the text and Lateral view in **C,** Photograph and **D,** Line drawing showing the anatomical features mentioned in the text.

Preopercle (pop). The preopercle is a stout and elongate bone in the ventral part of the suspensorium (Fig 6). It is firmly connected to the hyomandibula (hyo) by a bony suture dorsally and posteriorly. Anteriorly, the preopercle is separated from the quadrate (q) via a longitudinal groove which is the attachment of the synchondral joint. Laterally, the preopercle has a cylindrical tube-like process that reaches the hyomandibular blade (hyo.bl). In medial view, the preopercle has a large oval foramen, the mandibularis foramen (fm), at its antero-ventral part. In lateral view a circular foramen, the symplectic canal outer (upper) foramen (sc), is present in an oval depression near the preopercle-quadrate suture.

Hyomandibula (hyo). The bone is roughly rectangular and flat, and occupies half of the posterior dorsal part of the suspensorium (Fig 6). The hyomandibula connects dorsally to the sphenotic via the hyomandibular-sphenotic join. The latter is an oval condyle (ar.co.neuro) in the postero-dorsal corner of the hyomandibula that articulates with an oval facet in the sphenotic. The hyomandibula joins the preopercle and opercle postero-ventrally. The hyomandibular-opercle joint (opf) has a narrow projecting surface on the postero-ventral corner of the hyomandibula and a corresponding fossa on the medial side of the antero-dorsal corner of the opercle. The hyomandibular-preopercle joint is osseus and long. The hyomandibula articulates with the metapterygoid and quadrate anteriorly. The hyomandibular-quadrate joint is short and entirely synchondral, leaving a narrow groove, while the hyomandibular-metapterygoid joint is represented by a sinous line in the medial view and a zig-zag line in lateral view. Posterior to the hyomandibula-sphenotic joint there is a dorsally expanded thin hyomandibular blade (hyo.bl) that closely parallels but doesn't contact the skull roof margins of the sphenotic or pterotic. The hyomandibular process (hyo.pr) is well-developed, protrudes dorsally and is located anterior to, and separated by a notch from, the facet for articulation with the neurocranium (ar. co. neuro). Medially, the adductor hyomandibularis process (pah) for the attachment of the adductor mandibulae muscle projects posteriorly and is separated from the hyomandibular blade by a wide notch. The hyomandibula bears a large foramen for the ramus hyomandibularis of cranial nerve VII that is present ventral to the hyomandibular process. Laterally, the hyomandibula bears a well-developed longitudinal crest and broad net-shaped muscle scars for the levator arcus palatini muscle (clap). The antero-ventral part of the hyomandibula bears two small oval foramina; the mandibularis foramen (fm) near the hyomandibular-preopercle suture and the facial canal outer (lower) foramen (fc).

Metapterygoid (mto). The roughly rectangular metapterygoid is located dorsal to the quadrate and anterior to the hyomandibula (Fig 6). A part of the dorsal margin of the metapterygoid is broken. The metapterygoid-quadrate suture is interdigitated. The metapterygoid has a large notch anteriorly and is ornamented dorso-laterally with slightly developed pits and bars. The metapterygoid anterior process is absent.

Quadrate (q). The quadrate is a relatively stout and fan-shaped bone that is located ventral to the metapterygoid and anterior to the hyomandibula and preopercle (Fig 6). The articulating facet of the quadrate for the angulo-articular (ar.fa.ang.art) is present antero-ventrally and has a saddle shape. In medial view, the symplectic canal (sc) inner (lower) foramen, an opening for the mandibular branch of the ramus hyomandibularis, is large and oval and is situated at the same level as the articulating facet of the quadrate for the angulo-articular (ar.fa.ang.art). Laterally, the ventral border of the quadrate bears a prominent, thick crest to which a medial section of the adductor mandibulae muscle attaches.

#### Opercle

**Fig 7:**
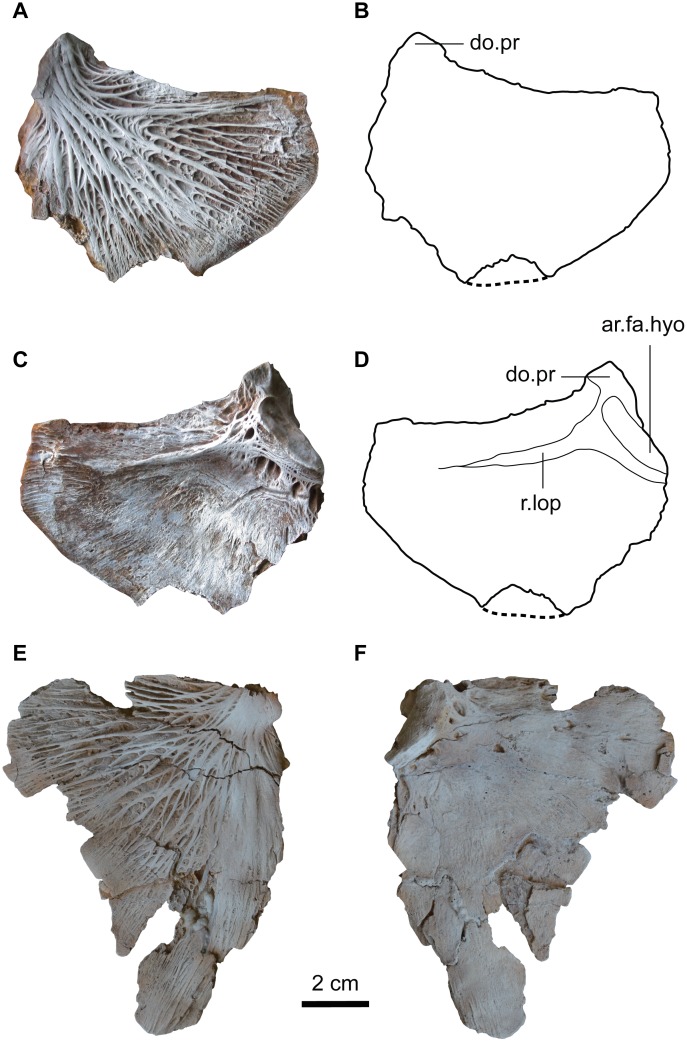
Left and right opercules of *Qarmoutus hitanensis* gen. et sp. nov. Dorsal view of the left opercle in **A,** Photograph and **B,** Line drawing showing the anatomical features mentioned in the text. Ventral view of the left opercle in **C,** Photograph and **D,** Line drawing showing the anatomical features mentioned in the text. Right operclular in **E,** Dorsal and **F,** Ventral views.

The opercle is flat, laminar and fan-shaped (subtriangular) bone posterior to the suspensorium. The left and right opercles are nearly complete and well-preserved in MUVP 58 aside from their antero-ventral margins (Fig 7). The ventral margin of the opercle is truncated and the anterior portion is not preserved. The posterior portion of the opercle is not well-developed and less rounded postero-dorsally with angular postero-dorsal corner and lacks the opercular spine. The dorsal margin is long and somewhat straight. In medial view, the facet for the hyomandibular-opercle joint (ar.fa.hyo) is situated antero-dorsally and has a shallow oval surface. A well-developed antero-dorsal process (do.pr) is present for the insertion of the dilatator operculi muscle. The opercle has a long and well-developed ridge (r.lop) originating at the articular facet for the hyomandibula but not reaching the posterior end of the bone for the insertion of the levator operculi muscle. In lateral view, the opercle is ornamented with strut-like radiating ridges branching and tapering from the articular facet for the hyomandibula to the margins of the bone.

#### Pectoral girdle

The left pectoral girdle was found articulated with the pectoral spine and very close to the neurocranium of MUVP 58. The three elements of the left pectoral girdle; posttemporo-supracleithrum, cleithrum and scapulo-coracoid are well-preserved.

**Fig 8:**
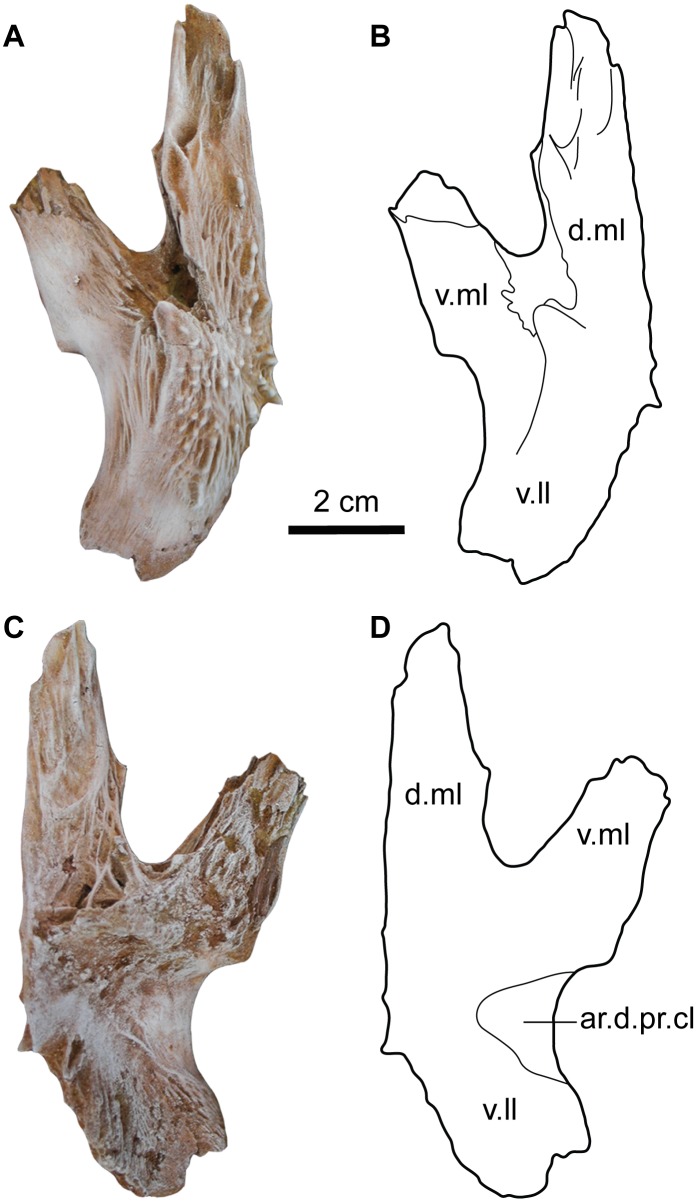
Left posttemporal-supracleithrum of *Qarmoutus hitanensis* gen. et sp. nov. Dorsal view in **A,** Photograph and **B,** Line drawing showing the anatomical features mentioned in the text. Ventral view in **C,** Photograph and **D,** Line drawing showing the anatomical features mentioned in the text.

Posttemporo-supracleithrum. This is the most dorsal element of the pectoral girdle bones, which connects the pectoral girdle to the neurocranium. In MUVP 58, the left posttemporo-supracleithrum was found isolated and not attached to the neurocranium (Fig 8). The bone is well-preserved and has three well-developed bony limbs. The dorso-medial limb (d.ml) is long and has an articulating surface that connects to an oval groove in the postero-lateral part of the extrascapular. The ventro-medial limb (v.ml) is shorter than the dorso-medial limb and its terminus has an articulating surface, which could be for a ligamentous attachment with the neurocranium. The ventro-lateral limb (v.ll) is broad and shows no bifurcation. The ventral surface of the ventro-lateral limb bears a smooth triangular articulating groove for the dorsal process of cleithrum (ar.d.pr.cl) and has an irregular surface posteriorly for the connective tissue that attaches with the Weberian apparatus. In dorsal view, the posttemporo-supracleithrum has a longitudinal bony channel between the two medial processes for the passage of the main lateralis sensory canal and is ornamented with tubercles that radiate from the center of the bone, which resembles that of the sphenotic.

**Fig 9:**
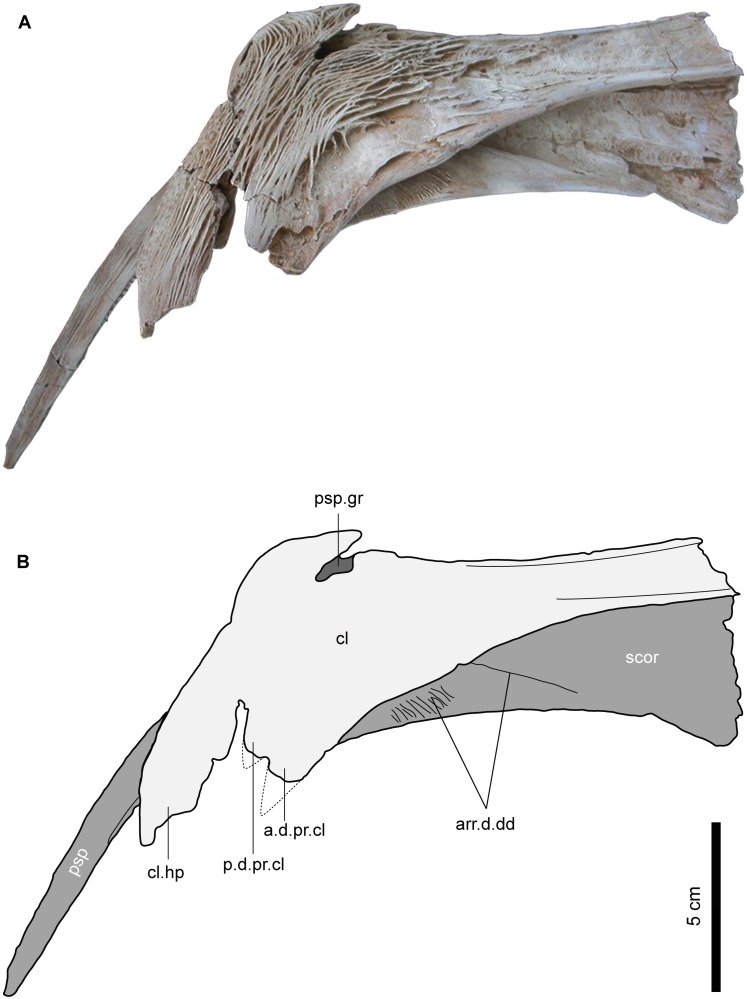
Left pectoral girdle of *Qarmoutus hitanensis* gen. et sp. nov. Dorsal view in **A,** Photograph and **B,** Line drawing showing the anatomical features mentioned in the text.

**Fig 10:**
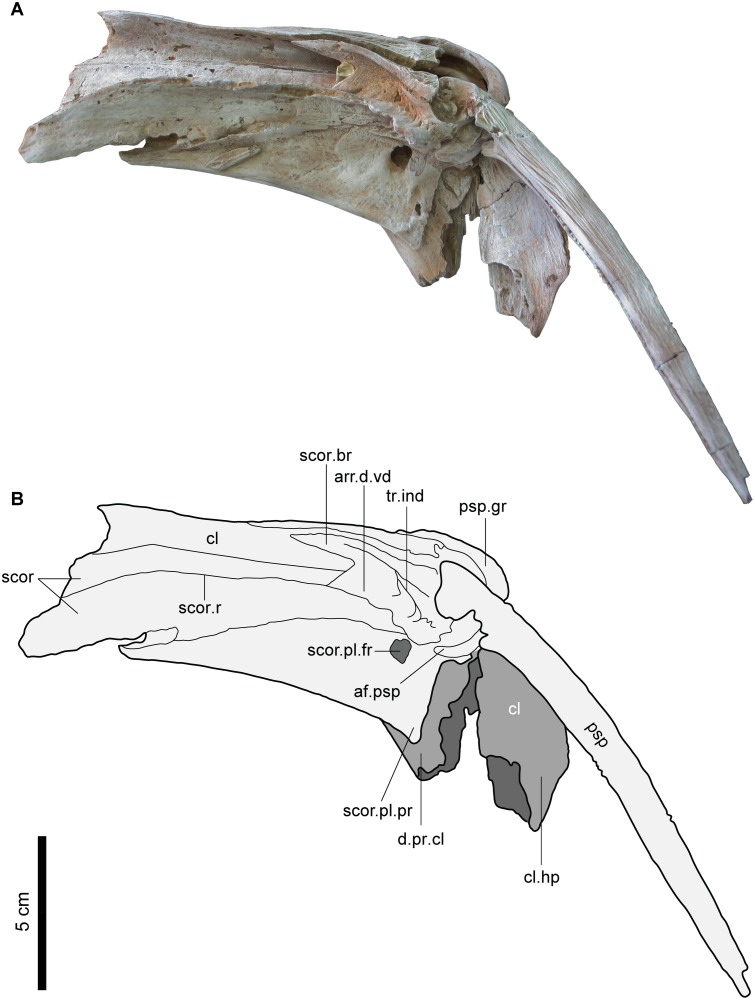
Left pectoral girdle of *Qarmoutus hitanensis* gen. et sp. nov. Ventral view in **A,** Photograph and **B,** Line drawing showing the anatomical features mentioned in the text.

Cleithrum (cl). The cleithrum is a large and robust element that constitutes a large part of the pectoral girdle and forms the posterior part of the branchial chamber (Figs 9 and 10). In MUVP 58, the cleithrum is elongate antero-posteriorly and has a wide posterior portion but is more narrow anteriorly. Postero-dorsally, the cleithrum has two prominent processes; the dorsal process of cleithrum (d.pr.cl) and the humeral process of cleithrum (cl.hp), both of which extend ventrally to border the pectoral fin spine groove (psp.gr). The dorsal process of the cleithrum articulates with the posttemporo-supracleithrum and is separated into the anterior dorsal process (a.d.pr.cl) and the posterior dorsal process (p.d.pr.cl). Ventro-laterally, the cleithrum bears a deep groove that accommodates the thick crescentic dorsal process of the proximal portion of the pectoral spine to form an antero-posteriorly mobile articulation. The antero-ventral border of the pectoral spine groove is bordered anteriorly by the scapulo-coracoid (Fig 10). The anterior border of the left cleithrum is short and abraded so the nature of its articulation with the right cleithrum is uncertain. The lateral and median margins of the cleithrum curl ventrally to form ridges, which create concave areas for muscle attachments (Fig 10). In ventral view, there is a crack running in the suture between the cleithrum and the scapulo-coracoid. In dorsal view, the posterior part of the cleithrum surface is ornamented with longitudinal, curved and reticulate ridges but, the most dorsal part of the humeral process of the cleithrum is ornamented with a network of tubercles like those seen on the pterotic, while the anterior part of the cleithrum lacks any ornamentation.

Scapulo-coracoid (scor). The scapulo-coracoid is an elongate bony plate sutured laterally to the cleithrum. In dorsal view, a large triangular part of the scapulo-coracoid is visible and possesses a rugose surface that is bounded anteriorly by a well-developed ridge for the origin of the dorsal division of the arrector dorsalis muscle (arr.d.dd) (Fig 9). The anterior margin of the left scapulo-coracoid is longer when compared to that of the cleithrum but it is abraded, which makes it difficult to determine the nature of articulation with the right scapulo-coraoid. In ventral view, the scapulo-coracoid has a somewhat rectangular shape with two raised and prominent structures; the scapulo-coracoid ridge (scor.r) and the scapulo-coracoid bridge (scor.br) both of which originate from the postero-lateral corner of the scapulo-coracoid and delimit a deep triangular indentation (tr.ind) and the ventral division of the arrector dorsalis muscle (arr.d.vd) (Fig 10). The latter is broad and occupies most of the area between the aforementioned structures. The scapulo-coracoid ridge is long and reaches the antero-median margin of the bone, while the scapulo-coracoid bridge is short and runs parallel to the antero-ventral margin of the cleithrum, creating a longitudinal groove. Postero-laterally, the cleithrum groove has a dorso-ventrally elongate articular facet (af.psp) that lodges in the base of the pectoral spine. The articular facet for the complex radial is obscured due to the articulation of the pectoral spine with the pectoral girdle. In the postero-lateral corner of the scapulo-coracoid, there is a short, broad and well-developed postero-lateral process (scor.pl.pr). The postero-lateral foramen of the scapulo-coracoid (scor.pl.fr), which is the passage for blood vessels and nerves, is large and circular and is situated roughly midway between the pectoral spine groove and the postero-lateral process. The foramen for the ventral condyle of the pectoral spine is absent, as well as the scapulo-coracoid spine and the mesocoracoid arch. In dorsal view, the scapulo-coracoid has a smooth surface and lacks any ornamentation.

**Fig 11:**
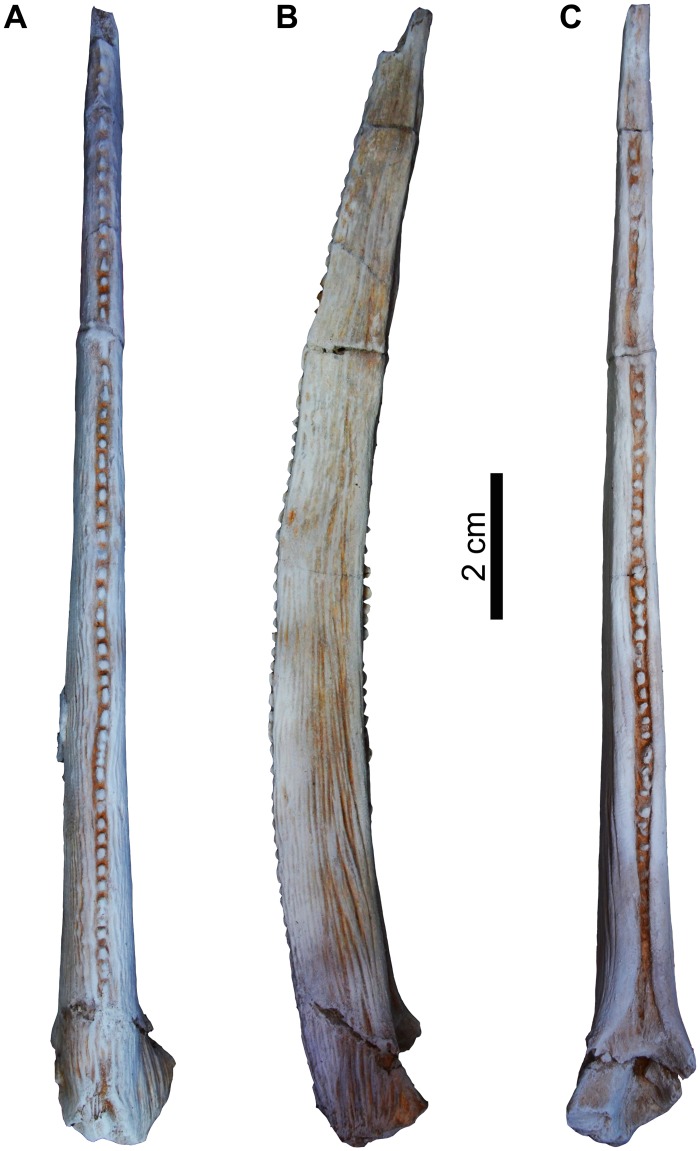
Left pectoral girdle spine of *Qarmoutus hitanensis* gen. et sp. nov. **A,** Anterior; **B,** Ventral and **C,** Posterior views.

**Fig 12:**
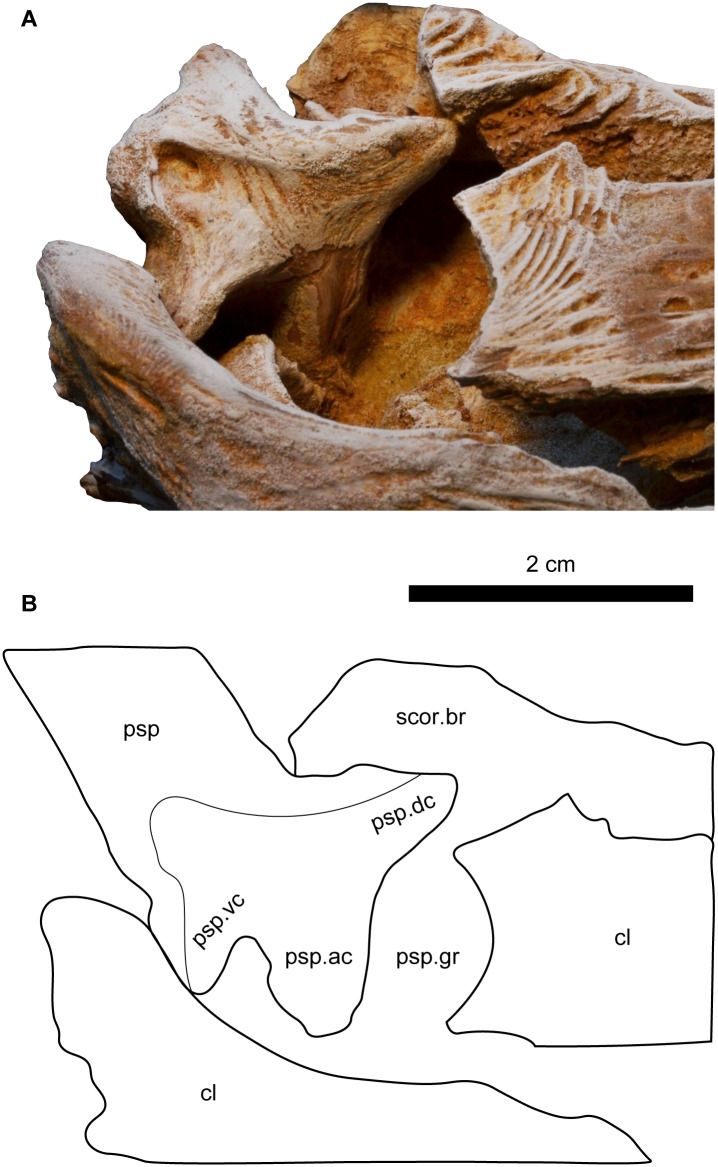
Close up view on the base of the left pectoral girdle spine of *Qarmoutus hitanensis* gen. et sp. nov. **A,** Dorsal and **B,** Line drawing showing the anatomical features mentioned in the text.

Pectoral fin spine (psp). The pectoral-fin spine is stout, complete and well-preserved, articulated with the pectoral girdle as in its natural position, and is missing only its most distal tip. The proximal base of the pectoral fin spine was found articulated with the pectoral girdle (Figs 11 and 12). The spine is long +165 mm in length, 11.55 mm in width and 6.56 mm in depth. The spine shaft is gently curved posteriorly, compressed dorso-ventrally and ornamented with longitudinal parallel ridges that extend from the base to the tip on the dorsal and ventral sides of the spine. Although the spine shaft tip is not completely preserved, it can be described as stout, sharp, not greatly elongate, and curved. In anterior view, the spine lacks the anterior ridge and bears continuous anterior dentations that start from about 1 cm above the spine base and terminates at the tip. The anterior dentations are 57 denticles that are low, rounded, erect and evenly spaced along the anterior edge.

In posterior view, there are 35 posterior denticles that are present in a midline groove, starting from about 10 mm from the proximal spine base to the spine tip. The posterior dentations are sharp, erect, regularly spaced, smoothly changing in size and some of them are bicuspid. There is a gap of no dentations on the distal one third of the spine. A deep small fossa is present on the posterior face of the spine just distal to the spine base, for articulation with the proximal radials. The proximal end of the pectoral spine has three well-developed condyles; anterior, ventral and dorsal condyles (Fig 12). The dorsal condyle is the largest condyle with a crescentic shape, ventral condyle is triangular, and the anterior condyle is thick and pointed medially.

#### Nuchal shield

The nuchal shield is a bony expansion of dermal bones that flank the dorsal fin. In MUVP 58, the nuchal shield is long and narrow and has an arrowhead shaped outline. It is composed of two distinct nuchal plates; the anterior nuchal plate and the posterior nuchal plate. The anterior and posterior nuchal plates are slightly raised medially with arched lateral portions ventrally. There is a longitudinal fracture that runs in the left lateral portion of the anterior nuchal plate and extends to the posterior margin of the posterior nuchal plate. The latter has also a longitudinal fracture in the right lateral portion that terminates at the suture line with the anterior nuchal plate. Anteriorly, there is a broad junction between the nuchal shield and the neurocranium, suggesting a relatively free movement for the nuchal shield. Ventrally, the two nuchal plates are fused with the neural spines of the subvertebral complex (Fig 4). The dorsal side of the nuchal shield has a tear-drop shape ornamentation that differs from the rest of dorsal skull roof ornamentation but similar to that of the parieto-supraoccipital process (Fig 2).

Anterior nuchal plate (anp). The anterior nuchal plate is well-preserved aside from minor cracks and minor damage in the left anterior lateral portion. The bone is robust and elongate with a narrow anterior portion and is longer than the posterior nuchal plate (Fig 2). In dorsal view, the anterior nuchal plate is roughly triangular in shape with a concave curved base. The anterior margin of the anterior nuchal plate is narrow and is articulated with the parieto-supraoccipital process via a sinuous broad connection that provides limited mobility for the anterior and posterior nuchal plate. Posteriorly, it has a long smooth convex junction with the posterior nuchal plate. The lateral margins of the anterior nuchal plate are relatively thick, straight and smooth. The ventral side of the anterior nuchal plate is concave and has a prominent longitudinal crest in its midpoint, which could be the articulation surface for the parapophysis of the complex centrum.

Posterior nuchal plate (pnp). The posterior nuchal plate is smaller than the anterior nuchal plate and has a butterfly shape (Fig 2). Anteriorly, the bone has two pointed projections that bound the posterior margin of the anterior nuchal plate. Posteriorly, the posterior nuchal plate has short and broad processes on either side of the cavity for the second dorsal spine. The articular surface of the posterior nuchal plate with the third pterygiophore is oval in shape and bears a well-developed rugose pattern, suggesting a tight connection between the two bones. The posterior and lateral margins of the posterior nuchal plate are smooth and curved. In ventral view, the posterior nuchal plate is arched and firmly articulates with the first petrygiophore.

#### Dorsal fins

**Fig 13:**
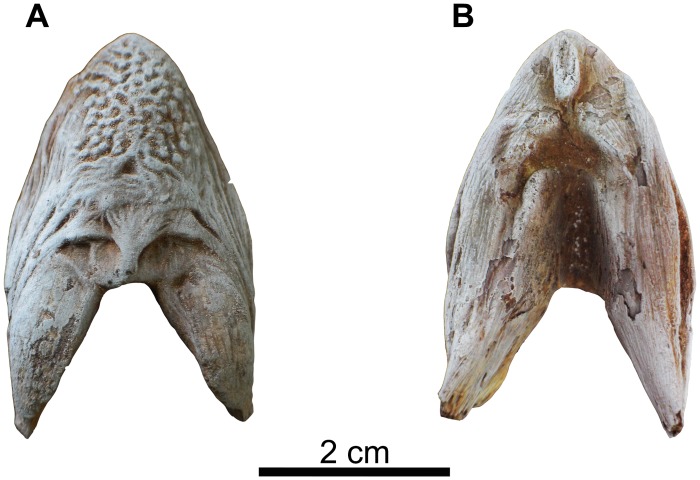
First dorsal spine of *Qarmoutus hitanensis* gen. et sp. nov. **A,** Anterior and **B,** Posterior views.

**Fig 14:**
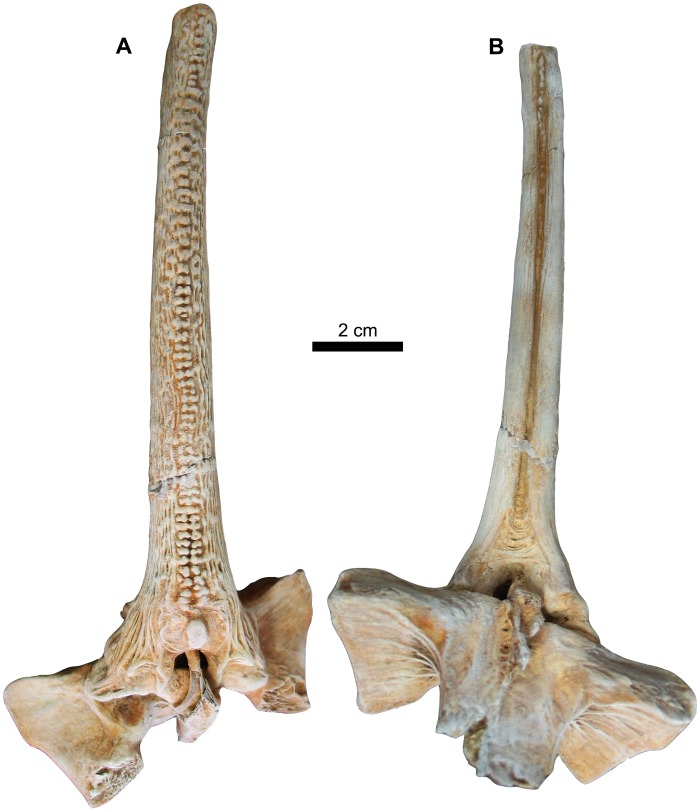
Second dorsal spine of *Qarmoutus hitanensis* gen. et sp. nov. **A,** Anterior and **B,** Posterior views.

**Fig 15:**
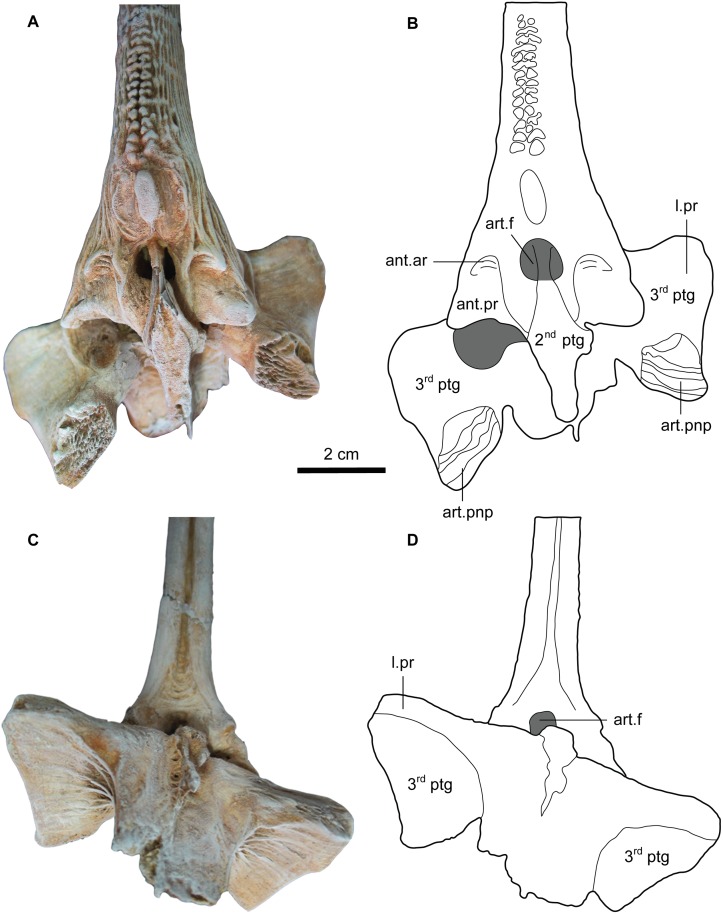
Second dorsal spine base of *Qarmoutus hitanensis* gen. et sp. nov. Anterior view in **A,** Photograph and **B,** Line drawing showing the anatomical features mentioned in the text. Posterior view in **C,** Photograph and **D,** Line drawing showing the anatomical features mentioned in the text.

**Fig 16:**
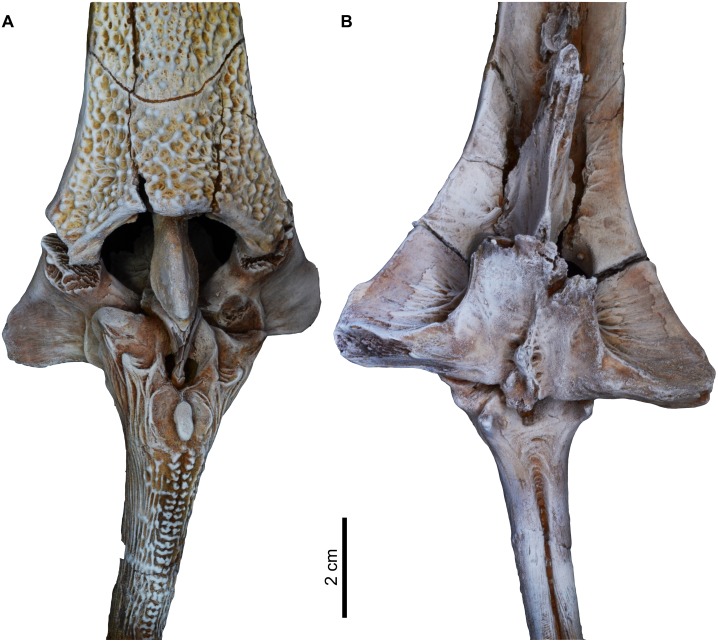
Articulated second dorsal spine with the posterior nuchal plate of *Qarmoutus hitanensis* gen. et sp. nov. **A,** Anterior and **B,** Posterior views.

The first and second dorsal fin spines and the pterygiophores are well-preserved in MUVP 58 (Figs 13, 14, 15 and 16). The second dorsal spine was found articulated with the second and third pterygiophores. The first pterygiophore is attached to the posterior nuchal plate (see above).

Pterygiophores (*ptg*). The pterygiophores, which are bony elements that support the dorsal fin, have three preserved different bones in MUVP 58. The first pterygiophore is a laminar bony plate that is strongly attached to the posterior nuchal plate and expanded laterally as very weakly-developed processes that contact the posterior processes of the posterior nuchal plate (Figs 4 and 16). Dorsally, the articulating surface of the first pterygiophore with the second pterygiophore is well-preserved and has a rhomboid shape. There is a wide gap between the first pterygiophore and the second dorsal spine for the accommodation of the first dorsal spine. The second and third pterygiophores are firmly sutured to each other. The second pterygiophore is fusiform with a smooth articulating surface and lacks the lateral expansions (Fig 15). The bone can only be seen from the dorsal view and has a median loop that interlocks with the articular foramen in the base of the second dorsal spine. The third pterygiophore has two wide and large lateral processes (l.pr), leading to a butterfly shape of the bone. In dorsal view, the right articular facet for the right anterior process of the second dorsal spine is exposed due to postmortem distortion, while the left articular facet fits perfectly with the left articular process of the second dorsal spine. The anterior articulating surfaces with the posterior nuchal plate (art.pnp) are visible anteriorly with a well-developed rugose surface and are separated by a broad bony plate. In ventral view, the posterior part of the lateral processes is lower when compared to the anterior part. There is a bony growth along the midpoint of the third pterygiophore.

First dorsal spine. The first dorsal spine is small, well-developed and arrowhead-shaped (Fig 13). The bone has distinctive lateral processes that are separated by a high arch, which accommodates the dorsal surface of the first pterygiophore in the gap between the nuchal shield and the second dorsal spine. In anterior view, the spine is concave and ornamented with longitudinal ridges laterally and small tubercles anteriorly. There are two small depressions separated by a short ridge at the ventral midpoint of the bone. In posterior view, the two lateral processes are separated by a longitudinal groove leading to a convex surface. There are three oval swellings near the tip of the spine. In lateral view, there is a narrow longitudinal groove on both sides.

Second dorsal spine. The second dorsal spine is complete and well-preserved and articulates with the second and third pterygiophores (ptg) as in its natural position with slight deviation due to postmortem distortion (Figs 14, 15 and 16). The width ranges from 12.3 at the tip to 38.4 mm at the base and the total length is 140 mm. The head of the spine is triangular in shape and has pointed well-developed lateral wings. Its anterior process (ant.pr) articulates with the articular facet of the third pterygiophore and lacks any ornamentation. Its smooth surface is bounded dorsally with the anterior articulations (ant.ar). In anterior view, the articular foramen (art.f) of the second dorsal spine is large and circular in shape and receives the median loop of the second pterygiophore. The second dorsal spine is ornamented with two separated rows of tubercles at the proximal base and fuses mid-shaft forming bicuspid tubercles that grade into a single row of tubercles at the distal end of the spine. There is a large oval tubercle at the proximal base of the second dorsal spine shaft. Laterally, the spine is straight, with no sign of curvature, and ornamented with closely spaced ridges that form longitudinal shallow grooves in the proximal base. In posterior view, there is a midline groove that starts as a triangular shape at the base and runs along the posterior surface with a single row of tubercles on its most distal end. The anatomical features of the ventral side of the second dorsal spine are obscured due to the articulation with the pterygiophores.

#### Weberian apparatus/ Subvertebral complex (svo)

The Weberian apparatus is a multifunctional complex mechanical device in otophysian fishes essentially improving the audition and consists of a double chain of ossicles joining the air bladder to the inner ear. In MUVP 58, the left portion of the Weberian apparatus is complete and well-preserved, while the right portion is missing some of its lateral elements due to weathering in the field (Fig 3). The bone is well-developed and has a butterfly shape with a long body. In lateral view, the first vertebra (Fig 4) is reduced to a rectangular-shaped centrum and is completely fused to the basioccipital anteriorly and the compound Weberian centrum posteriorly. The centra of the second to fourth vertebrae are indistinguishably fused, forming along and cylindrical compound vertebrae, while the fifth centrum is distinguishable and joins that compound centrum posteriorly. In ventral view, the most anterior part of the Weberian apparatus is overlapped by the basioccipital. The aortic canal (oac) is a large, circular and deep opening that is situated at the most anterior part of the subvertebral process and is delimited anteriorly by the posterior part of the basioccipital. The middle ventral part of the subvertebral complex shows a shallow groove with no elevated walls for the aortic canal (ac) that is separated from the opening of the aortic canal via a rugose area. Laterally, the complex centrum bears a large wing-like lateral lamina, the parapophysis of the fourth centrum (prp 4). The latter has no contact with the neurocranial bones and is divided laterally into strongly fused anterior and posterior processes. The anterior process (Müllerian ramus) is a thin and long prominent beak, but its connections are uncertain, while the posterior process is wide and shorter and appears behind the anterior process. On the dorsal surface of the fourth parapophysis there is a large oval depression that is covered by sediments, cleaning of which would make the fourth parapophysis vulnerable to breakage.

The tripus (tr) and its dorsal crest (dctr) form a feather shape in the lateral part of the Weberian apparatus and extend up to the fifth vertebra. The elongate mesial point of the tripus (the articular process) enters a deep concavity in the complex centrum. The transformator process (tptr), which connects the tripus to the os suspensorium, is present and has a cressentic shape. The dorsal crest of the tripus (dptr) is broad and long, has a smooth surface and lies below the parapophysis of the fourth centrum. There is a small gap between the parapophysis of the fourth centrum and the dorsal crest of the tripus. The latter articulates with the complex centrum in a deep cavity of the antero-lateral part of the parapophysis of the fourth centrum. The fifth centrum is elongate and nearly twice the length of an abdominal centrum and has a large (but broken) parapophysis (prp5). The centrum is strongly associated with the complex vertebra anteriorly and bears striations on its lateral surface.

#### Vertebrae

**Fig 17:**
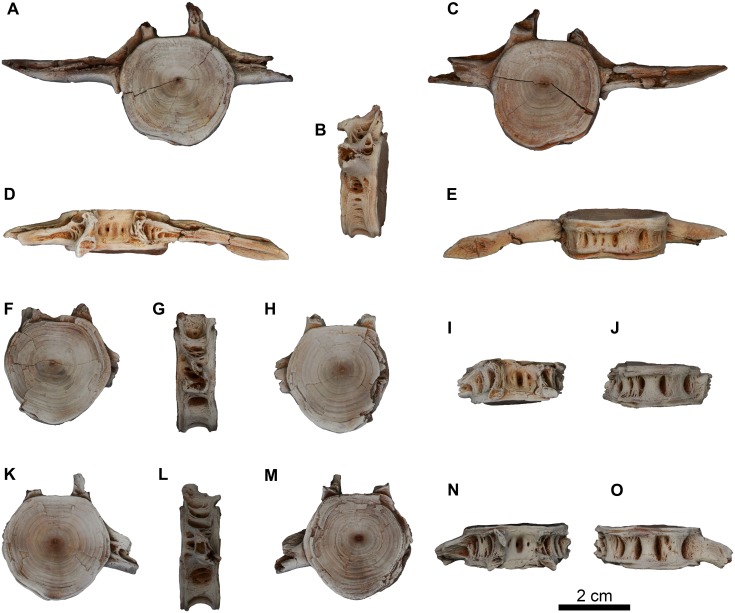
Abdominal vertebrae of *Qarmoutus hitanensis* gen. et sp. nov. Seventh abdominal vertebra of *Qarmoutus hitanensis* gen. et sp. nov. (MUVP 58). **A,** Anterior; **B,** Lateral; **C,** Posterior; **D,** Dorsal and **E,** Ventral views; eighth abdominal vertebra of *Qarmoutus hitanensis* gen. et sp. nov. (MUVP 58). **F,** Anterior; **G,** Lateral; **H,** Posterior; **I,** Dorsal and **J,** Ventral views; ninth abdominal vertebra of *Qarmoutus hitanensis* gen. et sp. nov. (MUVP 58). **K,** Anterior; **L,** Lateral; **M,** Posterior; **N,** Dorsal and **O,** Ventral views. For the lateral view, anterior is toward the left; for dorsal and ventral views anterior is toward the top of the page.

There are three isolated abdominal vertebrae, which are partially preserved (Fig 17) next to the MUVP 58 cranial elements. The centra of these vertebrae are slightly higher than wide. On the seventh abdominal vertebra (Fig 17A–17E), the articular surfaces of the centrum are somewhat rounded and are slightly wider dorsally than ventrally. The notochordal foramen is located more dorsally on the articular surfaces. In dorsal view, the dorsal surface of the seventh vertebra has a single longitudinal and shallow median depression that is bordered by low ridges and deep but small circular pits, all of which are bounded laterally by the neural arch bases. The latter continue laterally to connect the transverse processes via anterior and posterior projecting processes. These projecting processes delimit a triangular depression that bears circular pits. The transverse processes are strongly ossified, long and project from the dorso-lateral surfaces of the centrum at the same level of the notochordal foramen. The right transverse process is complete and long and has a pointed end. In lateral view, there are several deep oval articular pits that are separated by bony ridges. The ventral surface of the centrum has three median bony ridges bordered by deep circular pits.

The eighth and ninth abdominal vertebrae are identical (Fig 17F–17J and 17K–17O) and are as dorsoventrally tall as they are mediolaterally wide, with pentagonal articular surfaces. The notochordal foramen is located slightly dorsal to the midpoint of the articular surfaces. The dorsal surface has a deep oval depression that has a small circular pit in its floor. There are additional pits present lateral to this deep depression and are bounded by the neural arch bases. The dorsal parts of the neural arches are broken in the two vertebrae. The transverse processes are very weakly connected to the neural arch bases via very weakly developed projecting processes. None of the transverse processes are preserved aside from the base of the left transverse processes in both vertebrae. The preserved part of the transverse process is oriented posteriorly. In lateral view, the centrum has several pits dorsal to the transverse processes and a single large oval depression with two pits ventral to the transverse processes. In ventral view, there are three strong broad and well-developed ridges that extend antero-posteriorly and are bordered by large oval and deep circular pits.

## Comparisons and remarks

*Qarmoutus hitanensis* is the oldest known siluriform record of catfishes in the Fayum Depression (Priabonian, ~37 Ma), which previously was reported only from the upper Eocene and younger deposits in Egypt [21,22,25]. The first remains reported of catfishes were collected from the late Priabonian Qasr el-Sagha Formation of the Fayum Depression, northern Egypt [21]. The collected materials were a complete neurocranium of a new genus and species called *Fajumia schweinfurthi* (see Stromer,: Table 1, Figs 1 and 2 [21]) and a partial cranium and pectoral spine of a new genus and species named *Socnopaea grandis* (see Stromer,: Table.1, Figs 3 and 4[21]). Later, Peyer [22] described new materials for the previously described *Fajumia schweinfurthi* (see Peyer,: Table. 1 and 2, Table. 3 Figs a and b, Text-fig. 1–2 [22]) and *Socnopaea grandis* (see Peyer,: Table.3. Fig 3, Table. 4 Figs 2 and 4, Text-fig. 4 and 9 [22]). Peyer [22] also reported on an additional new genus and species (which would come to be named *Eopeyeria aegyptiaca*) and two new species (*Fajumia stromeri* and *Arius fraasi*). Peyer illustrated and described a neurocranium of *Fajumia stromeri* (see Peyer,: Table. 4 Fig 1, Text-fig. 3 [22]) in addition to a partial neurocranium and a series of associated vertebrae of *Eopeyeria aegyptiaca* (see Peyer,: Table. 5, Table. 6 Fig 1, Text-fig. 10 and 13 [22]). Our comparisons with these Egyptian Paleogene catfishes were by necessity made from the literature due to the inaccessibility of the type specimens.

**Fig 18:**
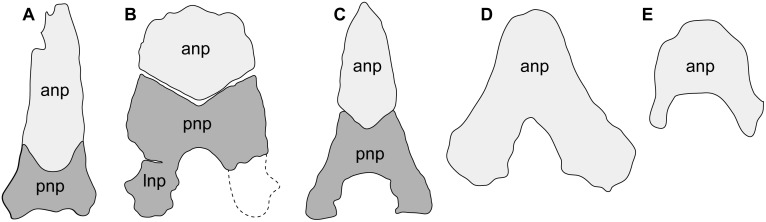
Comparative nuchal plate elements for the paleogene egyptian catfishes. **A,** *Qarmoutus hitanensis* gen. et sp. nov. (MUVP 58); **B,** *Fajumia schweinfurthi* (Stromer, [21] and Peyer, [22]); **C,** *Eopeyeria aegyptiaca* (Peyer, [22]); **D,** *Socnopaea grandis* (Stromer, [21] and Peyer, [22]) and **E,** *Arius fraasi* (Peyer, [22]).

The main differences between the Qasr el-Sagha Formation *Fajumia schweinfurthi* (the type species) and *Qarmoutus hitanensis* are the presence in the former of a broad and fan-shaped anterior portion of neurocranium; granular texture of the neurocranium, nuchal plates, pectoral girdle and opercle; a small horn on the anterolateral part of the mesethmoid; a short and broad parieto-supraoccipital process; and the lack of a connection between the medial groove of neurocranium and the parieto-supraoccipital. Moreover, the nuchal shield of *Fajumia schweinfurthi* differs from that of *Qarmoutus hitanensis* in having three nuchal plates, a hexagonal anterior nuchal plate with a jigsaw junction and V-shaped junctions with the parieto-supraoccipital and the median nuchal plate, respectively (Fig 18) and a square-shaped lateral nuchal plate. The ventral part of the neurocranium of *Fajumia schweinfurthi* shows also some differences such as the presence of two vomerine tooth plates, a broad basioccipital with distinct lateral expansions, the inclusion of the posterior part of the hyomandibular articular facet within the pterotic and an open aortic groove on the complex vertebrae. The suspensorium of *Fajumia schweinfurthi* differs from that of *Qarmoutus hitanensis* in having a narrow dorsally projecting hyomandibular blade and the symplectic canal in the quadrate. Aside from the ornamentation, the cleithrum of *Fajumia schweinfurthi* differs from *Qarmoutus hitanensis* in having a short and wide humeral process. In addition, the lower portion of the second dorsal spine of *Fajumia schweinfurthi* has three rows, while in *Qarmoutus hitanensis* it has two rows of tubercles.

*Qarmoutus hitanensis* also differs from the other species known from the Qasr el-Sagha Formation, *Fajumia stromeri*. The latter differs from *Qarmoutus hitanensis* in having a granular ornamentation on the dorsal surface; a concave anterior part, a small, oval medial groove of neurocranium bounded only by the frontals, a very small anterior fontanelle in the very anterior part of the neurocranium, broad and short parieto-supraoccipital process with a V shape junction with the anterior nuchal plate. Moreover, *Fajumia stromeri* lacks the vomerine tooth plates and has a very thick basioccipital and parasphenoid with lateral expansions of the basioccipital.

The neurocranium of *Socnopaea grandis* differs from *Qarmoutus hitanensis* in having a convex and broad anterior part; very small denticles parallel to the median groove ornamenting the cephalic shield bones, narrow anterior and posterior parts of the medial groove of neurocranium with a foraminal remnant for the posterior cranial fontanelle and a wide and long parieto-supraoccipital process. Moreover, *Socnopaea grandis* lacks the extrascapular bone, the pterotic is roughly square in shape, the sphenotic aligns with the frontal and the lateral ethmoid and the pterotic bone only expands laterally, the nuchal shield is triangular in shape with the anterior part oval in shape and smaller than the posterior and a concave-convex junction with the parieto-supraoccipital (Fig 18), very thick and broad parasphenoid and basioccipital and an interdigitating suture between the left and right pectoral girdles.

For *Eopeyeria aegyptiaca*, Peyer only reported the posterior part of the neurocranium with the nuchal plates, the second dorsal spine and the pectoral spine. *Eopeyeria aegyptiaca* differs from *Qarmoutus hitanensis* in having granular ornamentation on the dorsal surface of the neurocranium and the nuchal shield, a medium sized parieto-supraoccipital process with the same width in its anterior and posterior parts, a first nuchal plate that is spearhead-shaped (Fig 18) and has a concave and convex junction with the parieto-supraoccipital process, anterior and posterior processes of the parapophyses of the fourth vertebrae that are strongly fused, an oval opening for the aortic canal and the lack of the aortic tunnel in the vertebral complex. Moreover, the second dorsal spine is ornamented with denticles on its lateral surface, and the pectoral spine shaft is curved.

*Arius fraasi* differs from *Qarmoutus hitanensis* in that the neurocranium is concave anteriorly, ornamented with a granular texture, the medial groove of neurocranium is wide, narrow at its anterior part and is bounded by the frontals only. Moreover, the parieto-supraoccipital process is short and wide at its anterior part, the temporal fossa is present between the neurocranium and the posttempro-supracleithrum in the posterior part and one nuchal plate contacts the parieto-supraocciptal through a concave-convex junction (Fig 18).

## Results

### Parsimony analysis

**Fig 19:**
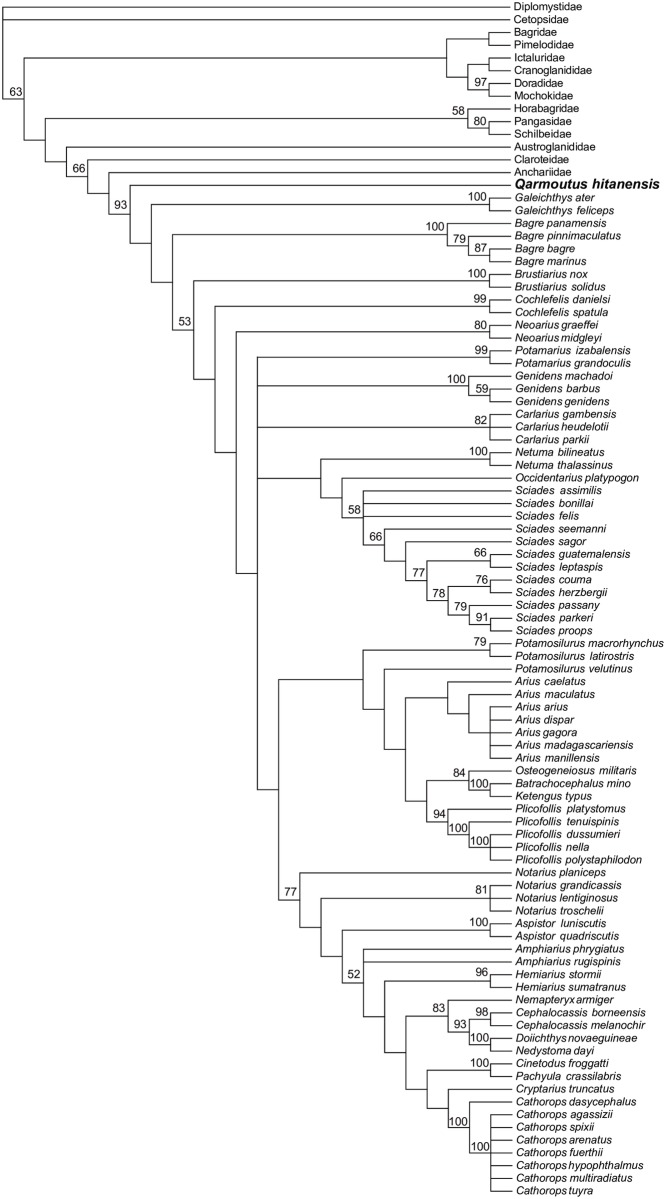
Parsimony analysis of Ariidae with no topological constraints, all characters equally weighted. Strict consensus of 1325 MPTs of 981 steps (CI excluding uninformative characters (CI) = 0.3247; Retention index (RI) = 0.8144; Rescaled consistency index (RCI) = 0.2723). Values above branches are bootstrap support values, based on 1,000 pseudoreplicates; values lower than 50 are not shown. Character support for nodes near *Qarmoutus* can be found in the main text.

Parsimony analysis with no topological constraints enforced, yielded *Qarmoutus* as a stem ariid, outside of a galeichthyine-bagreine-ariine clade (Fig 19). The resulting topology (tree length (TL) = 981; CI excluding uninformative characters (CI) = 0.3247; Retention index (RI) = 0.8144; Rescaled consistency index (RCI) = 0.2723) is largely similar to that of Marceniuk et al. ([18]; their Fig 29). The *Qarmoutus*-ariid clade is supported by four unambiguous synapomorphies [character 14, contact face between lateral ethmoid and frontal through two facets without the presence of a fenestra (state 2) = > contact face between lateral ethmoid and frontal through two facets that delimit a fenestra (state 3); character 31, form of medial groove of cranium deep with margins very conspicuous (state 1) = > form of medial groove of cranium shallow with margins not very conspicuous (state 0); character 71, subvertebral process indistinct or weakly developed (state 0) = > subvertebral process well developed (state 1); character 134, shape of dorsal crest of hyomandibular short and high (state 1) = > shape of dorsal crest of hyomandibular long and low (state 0)]. Support for the placement of *Qarmoutus* outside of crown Ariidae is weak (bootstrap <50%); the latter clade is supported by three unambiguous synapomorphies [character 13, shape of external posterior branch of lateral ethmoid depressed (state 1) = > shape of external posterior branch of lateral ethmoid columnar (state 0); character 68, size of otic capsule very small, restricted to prootic (state 0) = > size of otic capsule very large, limited by prootic, pterotic, and exoccipital (state 2); character 82, space between transcapular process and otic capsule very large (state 0) = > space between transcapular process and otic capsule moderately large (state 1)].

**Fig 20:**
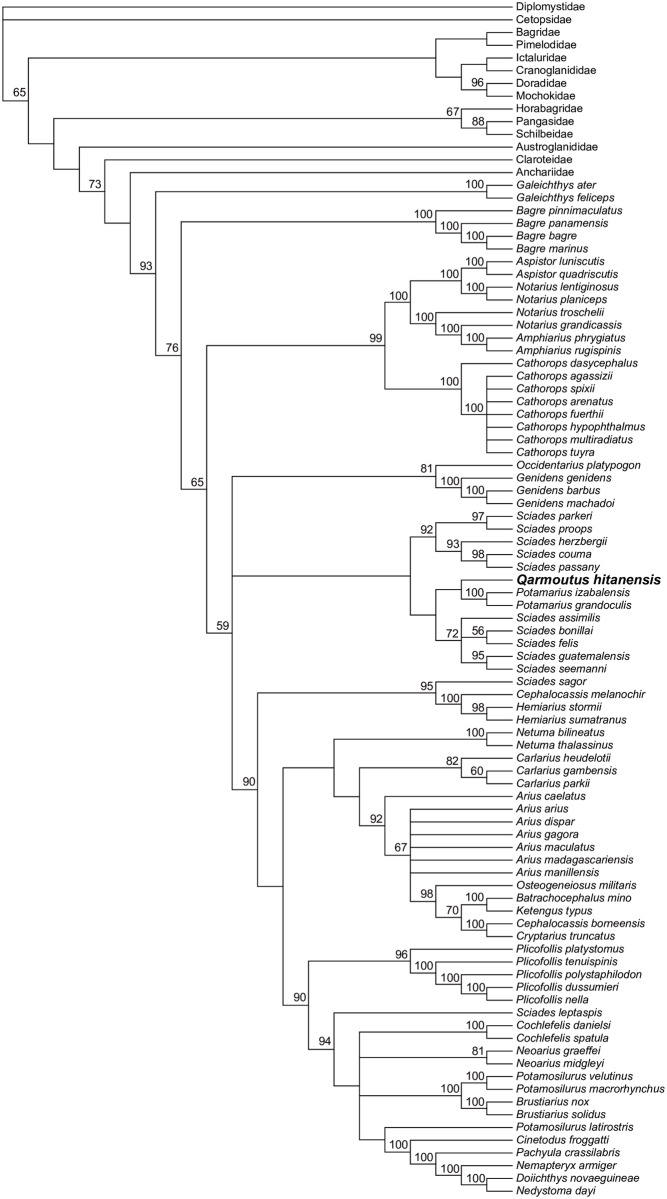
Parsimony analysis of Ariidae with molecular "scaffold" enforced, all characters equally weighted. Relationships were constrained to be consistent with nodes that were strongly supported (Bayesian posterior probabilities >0.95) in Betancur-R's molecular phylogenetic analysis of Ariidae. Strict consensus of 2844 MPTs of 1221 steps (CI excluding uninformative characters (CI) = 0.2601; Retention index (RI) = 0.7462; Rescaled consistency index (RCI) = 0.2005). Character support for nodes near *Qarmoutus* can be found in the main text.

Parsimony analysis, where extant species were constrained according to relationships reported by Betancur-R [38] recovered *Qarmoutus* nested deep within a *Sciades*/*Potamarius* clade, specifically as the sister taxon of the largely freshwater genus *Potamarius* from Central and South America, but without strong bootstrap support (Fig 20). The constrained parsimony analysis added 240 steps to overall tree length (TL = 1221) and resulted in a lower CI (0.2601), RI (0.7462), and RCI (0.2005), clearly indicating the presence of high levels of morphological homoplasy in the molecular tree. The *Potamarius*-*Qarmoutus*-*Sciades* clade was supported by six unambiguous synapomorphies [character 26, posterior cranial fontanel present (state 1) = > posterior cranial fontanel absent (state 0); character 28, epiphyseal bar conspicuous (state 0) = > epiphyseal bar indistinct (state 1); character 36, temporal fossa present (state 0) = > temporal fossa absent (state 1); character 68, size of otic capsule very large, limited by prootic, pterotic, and exoccipital (state 2) = > size of otic capsule moderate, limited by prootic, pterotic, and exoccipital (state 1); character 82, space between transcapular process and otic capsule moderately large (state 1) = > space between transcapular process and otic capsule very large (state 0); character 86, connection between posterior process of exoccipital and Müllerian ramus by ligaments (state 0) = > connection between posterior process of exoccipital and Müllerian ramus by suture (state 1). The *Qarmoutus* + *Potamarius* clade was supported by two unambiguous synapomorphies: [character 33, granulated (state 2) = > smooth or grooved and granulated (state 1) = > smooth or grooved (state 0); character 216, orientation of the second dorsal cleithral process posteriorly directed and parallel to posterior process (state 0) = > orientation of the second dorsal cleithral process dorsally directed and parallel to first dorsal process (state 1)].

### Bayesian analysis

**Fig 21:**
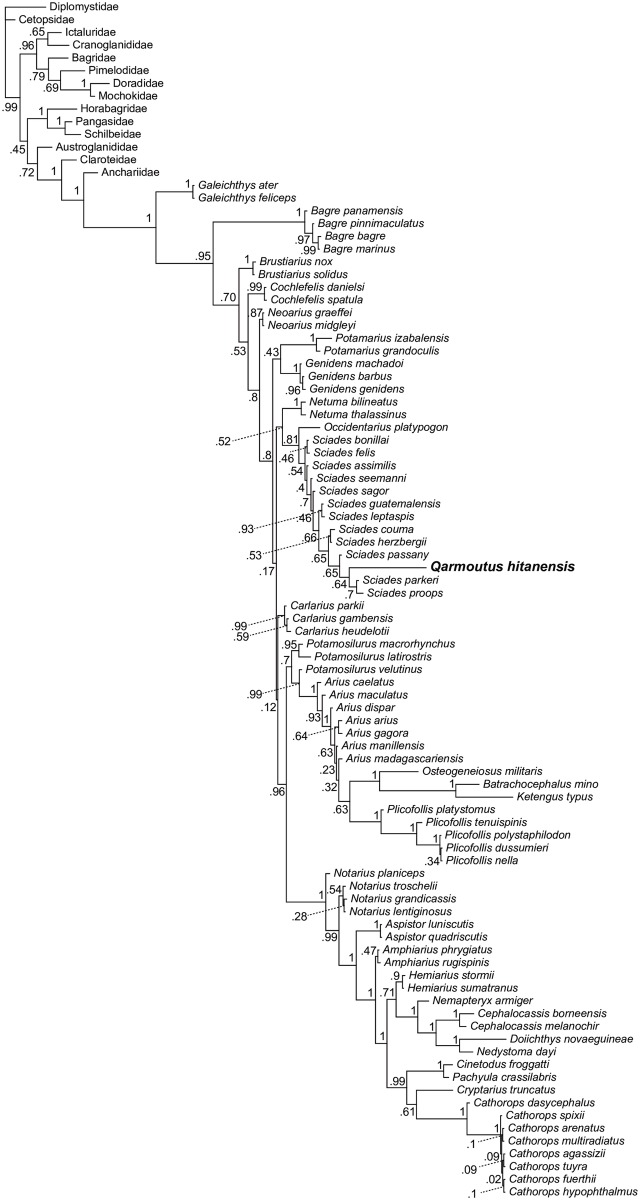
Bayesian phylogenetic analysis of Ariidae with no topological constraints. "Allcompat" consensus (majority-rule plus compatible groups) of 7,500 post burn-in trees retained following 10 million MCMC generations, sampled every 1,000 generations. Values above or below branches represent Bayesian posterior probabilities. Character support for nodes near *Qarmoutus* (parsimony optimization) is given in the main text.

Bayesian analysis with no topological constraints enforced recovered *Qarmoutus* deep within *Sciades* as the sister taxon of *Sciades parkeri* and *Sciades proops* (species from northern and northwestern South America; [44]) (Fig 21). Support for placement within *Sciades* is not very strong (posterior probabilities for supporting nodes range from of 0.40 to 0.70), and *Qarmoutus* is notable within this clade for having a particularly long terminal branch [~12 times longer than the longest terminal branch among *Sciades* species (i.e., for *Sciades proops*)]. Aside from the placement of *Qarmoutus*, the relationships among ariids are very similar to those recovered in the unconstrained parsimony analysis, and to those recovered by Marceniuk et al. [18]. Parsimony optimization of character state changes onto the Bayesian “allcompat” consensus indicates that the restricted clade containing *Qarmoutus*, *Sciades parkeri*, and *Sciades proops* is supported by two unambiguous synapomorphies [character 32, medial groove delimited exclusively by frontals (state 2) = > medial groove delimited mainly by frontals (state 1); character 208, shape of complex formed by anterior and median nuchal plates half-moon shaped (state 0) = > shape of complex formed by anterior and median nuchal plates shield-like (state 1)].

**Fig 22:**
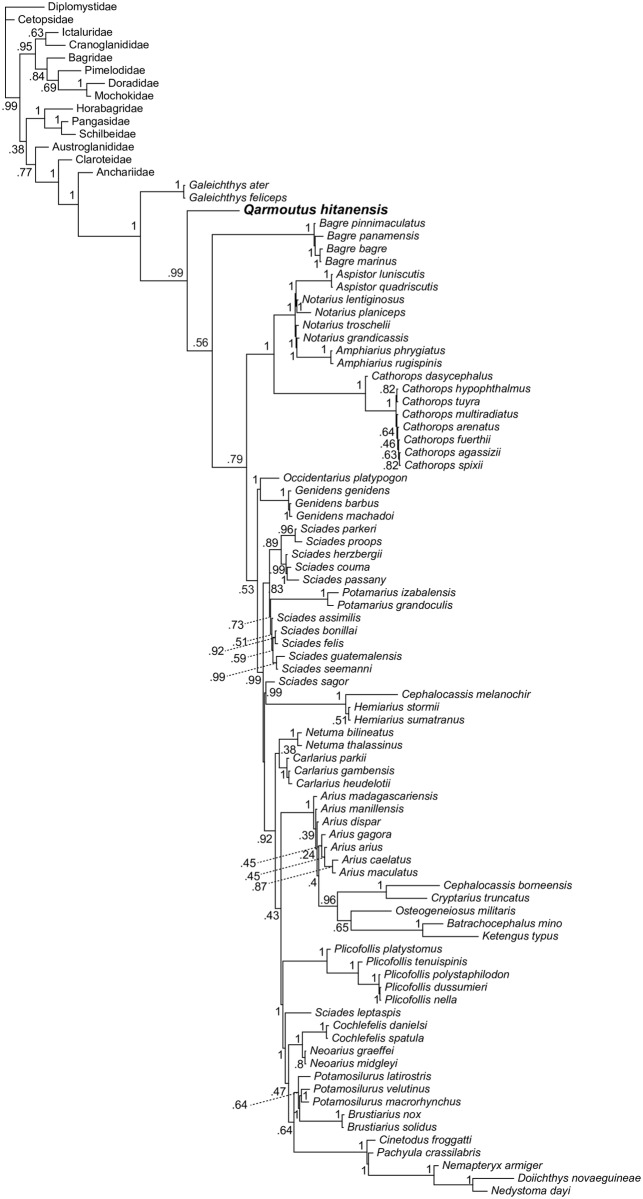
Bayesian phylogenetic analysis of Ariidae with molecular "scaffold" enforced (using partial constraints). Relationships were constrained to be consistent with nodes that were strongly supported (Bayesian posterior probabilities >0.95) in Betancur-R's molecular phylogenetic analysis of Ariidae. "Allcompat" consensus (majority-rule plus compatible groups) of 7,500 post burn-in trees retained following 10 million MCMC generations, sampled every 1,000 generations. Values above or below branches represent Bayesian posterior probabilities. Character support for nodes near *Qarmoutus* (parsimony optimization) can be found in the main text.

Bayesian analysis with topological constraints enforced placed *Qarmoutus* in a much more basal position within Ariidae, as a stem member of the bagreine-ariine clade, to the exclusion of Galeichthyinae (Fig 22). Support for the *Qarmoutus*-Bagreinae-Ariinae clade is strong (PP = 0.99), though support for Bagreinae+Ariinae to the exclusion of *Qarmoutus* is not (PP = 0.56). Parsimony optimization of character state changes onto the Bayesian “allcompat” tree indicates that the *Qarmoutus*+Bagreinae+Ariinae is supported by three unambiguous synapomorphies [character 24, anterior portion of anterior cranial fontanel not delimited by dorsal expansion of orbitosphenoid (state 0) = > anterior portion of anterior cranial fontanel partially or totally delimited by dorsal expansion of orbitosphenoid (state 1); character 69, enclosure of aortic canal absent (state 0) = > enclosure of aortic canal present (state 1); character 199, superficial ventral ossification of the Weberian apparatus not or only partially covering the aortic canal (state 0) = > superficial ventral ossification of the Weberian apparatus entirely covering the aortic canal (state 1)].

## Discussion and conclusion

Catfishes are a very diverse and species-rich group of teleostean fishes and are the most abundant freshwater fishes with members known to inhabit marine waters; ariids appear to have been ancestrally restricted to marine habitats but tolerance of freshwater has probably evolved multiple times independently [38]. Here we have compared the specimen MUVP 58, from the late Eocene sandstones of the Birket Qarun Formation, to other catfishes from the Paleogene of Egypt, and show that it belongs to a new genus and species, *Qarmoutus hitanensis*, that is not only the oldest of the Fayum catfishes but also the most complete one from the Birket Qarun Formation.

Ariidae and Plotosidae are the only two major clades of catfishes that have entered into marine habitats [11]. *Qarmoutus hitanensis* materials were unearthed from the shallow marine deposits of the upper Eocene Birket Qarun Formation exposed in the Wadi El-Hitan site of the Fayum Depression of northern Egypt. Most of them were articulated in their natural position or nearly in place, which suggests that the individuals died *in situ* with no postmortem transportation. Presence of the fossil whales *Basilosaurus isis* and *Dorudon atrox*, in addition to several sharks and sawfishes (e.g. *Hemipristis curvatus*, *Galeocerdo aegyptiacus*, *Misrichthys stromeri* and *Cretolamna twiggsensis*), in the same horizon alongside with *Qarmoutus hitanensis*, suggest a marine depositional setting.

As for the skull roof elements, the neurocranium of *Qarmoutus hitanensis* shows several synapomorphies that link it with the family Ariidae in having a well-developed basioccipital ventral process forming a cone-shaped projection [12,18], a superficial ventral ossification of the Weberian apparatus entirely covering the aortic canal [13,15,18], a fused basioccipital with the Weberian apparatus, anterior vertebral complex [45], a parasphenoid with a wing process [12], the Müllerian ramus [14], and lacks the mesocoracoid element in the pectoral girdle [15]. Dorsal and pectoral spines of Siluriformes are also distinguishable to a further extent to the family level. Gayet and Van Neer [46] identified the anatomical features that distinguish the ariid dorsal spine. The latter is constituted in its proximal part by two series of tubercles that regroup in a single series and have a massive and quadranglar articular head, a large median foramen with taller than wide outline, and well-developed lateral wings [46,45]. All of the aforementioned characters are present on the second dorsal spine of *Qarmoutus hitanensis*. The soft-tissue synapomorphies have been used by several authors [e.g.12,18] to define Ariidae, but they are not applicable on *Qarmoutus hitanensis*.

The contact relationship between the lateral ethmoid and the frontal is also an important synapomorphic character in Ariidae, which delimit a fenestra [14,15,18]. In *Qarmoutus hitanensis*, the fenestra delimited by the lateral ethmoid and the frontals is somewhat small as seen in *Sciades herzbergii*, *Sciades dowii* and *Plicofollis dussumieri*, not large as seen in *Cephalocassis melanochir* and *Cathorops agassizii*. Also having five pairs of bones connected with the parieto-supraoccipital (frontal, sphenotic, pterotic, extrascapular and the epioccipital) is a unique feature for Ariidae [45], but the epiocccipital in *Qarmoutus hitanensis* is not exposed dorsally. Another synapomorphic character for the family Ariidae is the largely developed lapillus that occupies the area corresponding to the prootic, pterotic and exoccipital, leading to a well-developed otic capsule [13,12,16,18]. In *Qarmoutus hitanensis* the otic capsule is weakly developed and small, restricted to the prootic, which indicates a small size of the otolith. A weakly differentiated otic capsule is also present in the ariid genus *Cathorops*.

The epioccipital bone that is in all of the ariid genera examined by Marceniuk et al. [18] has a posterior process, which is a synapomorphic character for Ariidae[15]. It is completely obscured in *Qarmoutus hitanensis* due to the attachment of the Weberian compound centrum posteriorly with the basioccipital. It is expected to be present entirely inside the posterior part of the neurocranium, which makes it difficult to examine this character in *Qarmoutus hitanensis*.

Most ariid genera have a subtrapezoidal and long anterior portion of the opercle [18], which is a diagnostic character for the family Ariidae. *Qarmoutus hitanensis* has also a subtrapezoidal but relatively short anterior portion of the opercle. This condition is observed in some ariid genera such *Cathorops*, *Cryptarius*, *Pachyula* and the species *Cephalocassis borneensis*. The transcapular process of *Qarmoutus hitanensis* is not preserved articulating with the basioccipital, however the space between transcapular process and the otic capsule could be predicted to be very large, a character seen in *Sciades* and *Occidentarius*. The previous character was described as small in Marceniuk and Menezes [12] and moderately developed in Marceniuk et al. [18] for distinguishing the ariid catfishes.

In most of Siluriformes, the anterior nuchal plate is differentiated from the posterior nuchal plate. However, the suture between the anterior and posterior nuchal plates is not recognizable in Anchariidae and almost all Ariidae except for *Bagre bagre* and *Galeichthys*. In. *Qarmoutus hitanensis*, the suture between the anterior and posterior nuchal plates is well developed.

Determination of *Qarmoutus*’ position in ariid phylogeny is complicated by the strong conflict between the morphological and molecular evidence for ariid relationships that has been revealed by the morphological and molecular studies of Marceniuk et al. [18] and Betancur-R [38], respectively. Among other discrepancies, multiple extant ariid genera (for instance *Brustiarius*, *Cochlefelis*, and *Neoarius*) are placed in radically different positions (either phylogenetically basal within Ariidae based on morphological data, or deeply nested within an Australian-New Guinean ariid clade based on molecular data), depending on which type of data are being considered. However the highly variable positions of *Qarmoutus* in our phylogenetic analyses are not due simply to whether morphological data are constrained to fit the molecular tree or not, but also method of phylogenetic reconstruction (i.e., Bayesian versus parsimony)—that is, *Qarmoutus* is placed basal in both constrained (Bayesian) and unconstrained (parsimony) analyses, and deeply nested in both constrained (parsimony) and unconstrained (Bayesian) analyses.

Since *Qarmoutus* is effectively placed in only two different positions (either near the base of Ariidae (Figs 19 and 22), or deep within a clade of New World *Sciades* (with or without *Potamarius*) (Figs 20 and 21), it is worth evaluating which of these radically different results is more likely, given other considerations and the nature of the support for these alternative placements. There are two primary lines of evidence that we consider important in this regard. First is *Qarmoutus*’ great antiquity; its late Eocene age would be consistent with a very basal placement in Ariidae given Betancur-R’s [17] molecular divergence estimate of ~40 Ma for the origin of bagriines+ariines, Alternatively, if the genus is indeed nested deeply within “*Sciades*”, several species in that genus would require ghost lineages extending back to the late Eocene, if not much earlier, and would predict an ancient origin for Ariinae that would be wildly inconsistent with available molecular divergence estimates and the succession of ariid species in the fossil record. Second, in the unconstrained Bayesian analysis, *Qarmoutus* can only be accommodated within *Sciades* by being placed along a very long terminal branch—therefore not only requiring the extensive ghost lineages mentioned above, but also requiring that this very ancient ariid would already, in the late Eocene, be exceptionally autapomorphic. Long ghost lineages and exceptional autapomophies are also indicated when *Qarmoutus* is analyzed in the constrained parsimony analysis, and recovered as the sister taxon of *Potamarius*. This placement requires a total of 15 unambiguous autapomorphies along the terminal branch leading to *Qarmoutus*, whereas *Potamarius* species are reconstructed as having only two (*Potamarius grandoculis*) and five (*Potamarius izabalensis*) autapomorphies.

A basal placement for *Qarmoutus*, either as a stem ariid or as a stem ariine, would be of biogeographic interest given Betancur-R’s argument [17] that galeichthyines are likely to have been ancestrally African. Whether *Qarmoutus* is placed as a stem ariid, as in the unconstrained parsimony analysis, or as a stem member of the bagreine-ariine clade, as in the constrained Bayesian analysis, the African Galeichthyinae and *Qarmoutus* would be paraphyletic with respect to non-African ariids and would provide support (albeit weak) for the African origin of Ariidae. However, given the ambiguity of our phylogenetic results and the clear potential for morphological homoplasy among living and extinct ariids, it appears that additional molecular sequences from extant taxa and improved sampling of the early fossil record of Ariidae will be required to ultimately place living and extinct members of this family into a more robust phylogenetic framework.

## Supporting information

S1 Appendix Modified character set of Marceniuk et al. [18] employed in phylogenetic analyses. (DOCX)

S2 Appendix Character state matrix. Character matrix of 230 characters for the Ariidae species and outgroups.
(PDF)

## References

[1] Andrews CW. A descriptive catalogue of the Tertiary Vertebrata of the Fayum, Egypt; based on the collection of the Egyptian Government in the Geological Museum, Cairo, and on the collection in the British Museum (Natural History). Trustees of the British Museum (Natural History) 1904; 324 pp.

[2] Gingerich PD, Smith BH, Simons EL. Hind limbs of Eocene *Basilosaurus*: evidence of feet in whales. Science. 1990; 229: 154–157. 10.1126/science.249.4965.154 17836967

[3] Uhen MD. Form, function, and anatomy of *Dorudon atrox* (Mammalia, Cetacea), An archaeocete from the Middle to Late Eocene of Egypt. University Michigan Papers Paleontol. 2004; 34: 1–79.

[4] Gingrich PD, Abd Elshafy E, Metwally MHM, Zalmout IS and Antar MS. Paleoenvironmental and taphonomic assessment of Eocene marine fauna of Wadi El- Hitan and Siwa areas, Egypt. Egypt. J Paleontol. 2012; 11: 171–190.

[5] Brochu CA and Gingerich PD. New tomistomine crocodylian from the Middle Eocene (Bartonian) of Wadi Hitan, Fayum province, Egypt. University of Michigan Contrib. Museum Paleontol. 2000; 30(10): 251–268.

[6] Domning DP, Gingerich PD. *Protosiren smithae*, new species (Mammalia, Sirenia), from the late Middle Eocene of Wadi Hitan, Egypt. University of Michigan Contrib. Museum Paleontol. 1994; 29: 69–87.

[7] Zalmout IS and Gingerich PD. Late Eocene sea cows (Mammalia, Sirenia) from Wadi Al Hitan in the Western Desert of Fayum, Egypt. University Michigan Papers Paleontol. 2012; 37: 1–175.

[8] Underwood CJ, Ward DJ. New hemigaleid shark from the late Eocene of Wadi Al-Hitan, Egypt. J Vertebr Paleonto. 2011; 31: 707–711.

[9] Underwood CJ, Ward DJ, King C, Antar MS, Zalmout IS, Gingerich PD. Shark and ray faunas in the Late Eocene of the Fayum, Egypt. Proceedings of the Geologists’ Association. 2011; 122:47–66.

[10] Nelson JS. Fishes of the World. 4th ed Hoboken (New Jersey, USA): John Wiley and Sons 2006; 601 pp.

[11] Baras E, Lalèyè P. Ecology and behavior of catfishes In Arratia G, Kapoor BG, Chardon M, Diogo R. et al (eds): Catfishes. Science Publishers, Inc., Enfield, NH, USA; 2003 525–579.

[12] Marceniuk AP, Menezes NA. Systematics of the family Ariidae (Ostariophysi, Siluriformes), with a redefinition of the genera. Zootaxa. 2007; 1416: 1–126.

[13] Mo T. Anatomy, relationships and systematics of the Bagridae (Teleostei: Siluroidei) with a hypothesis of siluroid phylogeny. Koenigstein, Koeltz 1991; 1–216.

[14] De Pinna MCC. Higher-level phylogeny of Siluriformes (Teleostei: Ostariophysi), with a new classification of the order. Unpublished PhD Dissertation, City University of New York, New York. 1993; 1–482.

[15] Kailola PJ. A phylogenetic exploration of the catfish family Ariidae (Otophysi; Siluriformes). The Beagle, Records of the Museums and Art Galleries of the Northern Territory. 2004; 20: 87–166.

[16] Betancur-R R, Acero PA, Bermingham E, Cooke R. Systematics and biogeography of New World sea catfishes (Siluriformes: Ariidae) as inferred from mitochondrial, nuclear, and morphological evidence. Molecular Phylogenetics and Evolution. 2007; 45: 339–357. 10.1016/j.ympev.2007.02.022 17475516

[17] Betancur-R R, Armbruster JW. Molecular clocks provide new insights into the evolutionary history of galeichthyine sea catfishes. Evolution. 2009; 63: 1232–1243. 10.1111/j.1558-5646.2009.00640.x 19187251

[18] Marceniuk AP, Menezes NA, Britto MR. Phylogenetic analysis of the family Ariidae (Ostariophysi: Siluriformes), with a hypothesis on the monophyly and relationships of the genera. Zoological Journal of the Linnean Society. 2012; 165: 534–669.

[19] White E. Eocene fishes from Nigeria. Bulletin of the Geological Survey of Nigeria.1926; 10: 1–87.

[20] Gayet M, Meunier FJ. Palaeontology and Palaeobiogeography of Catfishes In Arratia G, Kapoor BG, Chardon M, Diogo R. et al (eds): Catfishes. Science Publishers, Inc., Enfield, NH, USA; 2003 491–522.

[21] Stromer E. Nematognathi aus dem Fajûm und dem Natronthale in Aegypten. N. Jb. Geol. Paläont. Mh. 1904;1: 1–17.

[22] Peyer B. Die Welse des Ägyptischen Alttertiärs nebst einer kritischen Übersicht über alle fossilen Welse. Abhandlungen der Bayerischen Akademie der Wissenschaften, Mathematisch-naturwissenshaftliche Abteilung. 1928; 32: 1–61.

[23] Whitley GP. New sharks and fishes from Western Australia. Part 3. Australian Zoologist. 1947; 11, 129–150.

[24] Ferraris CJ. Checklist of catfishes, recent and fossil (Osteichthyes: Siluriformes), and catalogue of siluriform primary types. Zootaxa. 2007; 1418: 1–628.

[25] Greenwood PH. Review of Cenozoic freshwater fish faunas in Africa. Annals of the Geological Survey of Egypt. 1974; 4: 211–232.

[26] Murray AM. Late Eocene and early Oligocene teleost and associated ichthyofauna of the Jebel Qatrani Formation, Fayum, Egypt. Palaeontology. 2004; 47: 711–724.

[27] Murray AM, Cook P, Attia Y, Chatrath P, Simons E. A freshwater ichthyofauna from the late Eocene Birket Qarun Formation, Fayum, Egypt. J Vertebr Paleontol. 2010; 30: 665–680.

[28] Otero O, Pinton A, Cappetta H, Adnet S, Valentin X, Salem M, et al A fish assemblage from the Middle Eocene from Libya (Dur At-Talah) and the earliest record of modern African fish genera. PLoS ONE. 2015; 10(12): e0144358 10.1371/journal.pone.0144358 26674637 PMC4684465

[29] Abdel-Fattah ZA, Gingras MK, Pemberton SG. Significance of hypburrow nodule formation associated with large biogenic sedimentary structures in open-marine bay siliciclastics of the Upper Eocene Birket qarun Formation, Wadi El-Hitan, Fayum, Egypt. Sedimentary Geology. 2011; 233: 111–128.

[30] Strougo A, Faris M, Haggag M, Abul-nasr R, Gingerich PD. Planktonic foraminifera and calcareous nannofossil biostratigraphy through the Middle to Late Miocene transition at Wadi Hitan, Fayum Province, Egypt. University of Michigan Contrib. Museum Paleontol. 2013; 32: 111–138.

[31] Gingerich PD. Marine mammals (Cetacea and Sirenia) from the Eocene of Gebel Mokattam and Fayum, Egypt: stratigraphy, age, and paleoenvironments. University Michigan Papers Paleontol. 1992; 30: 1–84.

[32] Haggag MA. *Globigerina pseudoampliapertura* Zone, a new late Eocene planktonic foraminiferal zone (Fayoum area, Egypt). N. Jb. Geol. Paläont. Mh. 1990; 5: 295–307.

[33] Elewa AMT, Omar AA, Dakrory AM. Biostratigraphical and paleoenvironmental studies on some Eocene ostracodes and forminifers from the Fayum depression, western desert, Egypt. Egypt J Geol 1998; 42 (2): 439–469.

[34] Anan T, El Shahat A. Provenance and sequence architecture of the Middle–Late Eocene Gehannam and Birket Qarun formations at Wadi Al Hitan, Fayum province, Egypt. J Afr Earth Sci. 2014; 100: 614–625.

[35] Abdel-Fattah ZA, Gingras MK, Caldwell MW, Pemberton SG. Sedimentary environments and depositional characteristics of the Middle to Upper Eocene whale-bearing succession in the Fayum Depression, Egypt. Sedimentology. 2010; 57: 446–476.

[36] Longbottom AE. A new species of the catfish *Nigerium* from the Palaeogene of the Tilemsi valley, Republic of Mali. Palaeontology. 2010; 53: 571–594.

[37] Vanscoy T, Lundberg JG, Luckenbill KR. Bony ornamentation of the catfish pectoral-fin spine: comparative and developmental anatomy, with an example of fin-spine. Proceedings of the Academy of Natural Sciences of Philadelphia2015; 164: 177–212.

[38] Betancur-R R. Molecular phylogenetics supports multiple evolutionary transitions from marine to freshwater habitats in ariid catfishes. Molecular Phylogenetics and Evolution. 2010; 55: 249–258. 10.1016/j.ympev.2009.12.018 20045737

[39] Swofford DL. PAUP* Phylogenetic Analysis Using Parsimony (*and Other Methods), Version 4 Sunderland, MA: Sinauer Associates 1998.

[40] Ronquist F, Teslenko M, van der Mark P, Ayres DL, Darling A, Höhna S, Larget B, et al MrBayes 3.2: efficient Bayesian phylogenetic inference and model choice across a large model space. Syst Biol. 2012; 61(3): 539–542. 10.1093/sysbio/sys029 22357727 PMC3329765

[41] Fink SV, Fink WL. Interrelationships of the ostariophysan fishes (Teleostei) in Stiassny MLJ, Parenti LR, Johnson GD. (eds.), Interrelationships of Fishes. Academic Press, New York 1996; 209–249

[42] Bleeker P. Atlas ichthyologique des Indes Orientales Néêrlandaises, publié sous les auspices du Gouvernement colonial néêrlandais Tome II. Siluroïdes, Chacoïdes et Hétérobranchoïdes. Amsterdam 1862; 112 pp.

[43] Arratia G, Gaye M. Sensory canals and related bones of Tertiary siluriform crania from Bolivia and North America and comparison with recent forms, J Vertebr Paleontol. 1995; 15(3): 482–505.

[44] Froese R, Pauly D. FishBase. 2016 World Wide Web electronic publication www.fishbase.org.

[45] Otero O, Pinton A, Mackaye HT, Likius A, Vignaud P, Brunet M. Fishes and palaeogeography of the African drainage basins: Relationships betweenChad and neighbouring basins throughout the Mio-Pliocene, Palaeogeography, Palaeoclimatology, Palaeoecology. 2009; 274: 134–139.

[46] Gayet M, Van Neer W. Caract`eres diagnostiques des ´epines de quelques silures africains. J Africain Zoology-Revue de Zoologie Africaine. 1990 104;241–252.

