# Supplementary material for: A new genus and species of marine catfishes (Siluriformes; Ariidae) from the upper Eocene Birket Qarun Formation, Wadi El-Hitan, Egypt
Source: PLoS One. 2017 Mar 1;12(3):e0172409. doi: 10.1371/journal.pone.0172409 (PMC5332075; doi:10.1371/journal.pone.0172409)
Supplement: S1 Appendix — (DOCX) [file pone.0172409.s001.docx]

**S1 Appendix. Modified character set of Marceniuk et al. [18] employed in phylogenetic analyses.**

**1. Form of mesethmoid medial notch:** large and shallow (state 0); narrow and deep (state 1)

**2. Mesethmoid median portion (minimally connected):** very narrow (state 0); moderately wide (state 1); very wide (state 2).

**3. Fenestra delimited by mesethmoid and lateral ethmoid:** absent (state 0); present (state 1).

**4. Fenestra delimited by mesethmoid and lateral ethmoid:** small, not filled with cartilage (state 0); large, filled with cartilage (state 1).

**5. Form of mesethmoid posterior horn:** depressed, very wide and short (state 0); tubular, narrow, and elongate (state 1).

**6. Width of posterior branches of mesethmoid:** wide (state 0); narrow (state 1).

**7. Relationships between posterior branches of mesethmoid:** divergent posteriorly (state 0); posterior branches parallel throughout their entire extension (state 1).

**8. Length and contribution of posterior branches of mesethmoid to delimitation of anterior cranial fontanel (maximally connected):** short, delimiting anteriorly about one quarter of length of anteriocranial fontanel (state 0); moderately long, delimiting between one quarter and half of length of anterior cranial fontanel (state 1); very long, delimiting half of length of anterior cranial fontanel (state 2).

**9. Shape of lateral ethmoid:** not expanded (state 0); expanded (state 1).

**10. Lateral horn of lateral ethmoid:** inconspicuous (state 0); conspicuous (state 1).

**11. Shape of lateral horn of lateral ethmoid:** slightly compressed and acute (state 0); very compressed and spatulate (state 1).

**12. Length and disposition of lateral horn of lateral ethmoid (minimally connected):** short and laterally orientated (state 0); moderately long and lateroposteriorly orientated (state 1); long and posteriorly orientated (state 2).

**13.** **Shape of external posterior branch of lateral ethmoid:** columnar (state 0); depressed (state 1).

**14.** **Contact face between lateral ethmoid and frontal (minimally connected):** absent (state 0); through a single facet (state 1); through two facets without the presence of a fenestra (state 2); through two facets that delimit a fenestra (state 3).

**15.** **Composition of bony bridge formed by lateral ethmoid and frontal (minimally connected):** lateral ethmoid is main component (state 0); lateral ethmoid and frontal are equally represented (state 1); frontal is main component (state 2).

**16.** **Thickness of bony bridge formed by lateral ethmoid and frontal:** thick to moderately thick (state 0); very thin (state 1).

**17. Size of fenestra delimited by lateral ethmoid and frontal (minimally connected):** very small or indistinct (state 0); moderately large (state 1); very large (state 2).

**18. Position of nasal in relation to mesethmoid:** parallel (state 0); perpendicular (state 1)

**19. Shape of nasal (maximally connected):** shaped like a hockey club (state 0); shape not well-defined (state 1); fan shaped (state 2).

**20. Nasal anterior curvature:** little pronounced (state 0); very pronounced (state 1).

**21. Bony blade anteriorly connecting nasal tubules:** absent (state 0); present (state 1).

**22. Relationship between lachrymal-antorbital and frontal:** coalesced or only partially differentiated (state 0); distinct from each other (state 1)

**23. Frontal mesial laminar projection:** absent (state 0); present (state 1).

**24. Anterior portion of anterior cranial fontanel:** not delimited by dorsal expansion of orbitosphenoid (state 0); partially or totally delimited by dorsal expansion of orbitosphenoid (state 1).

**25. Bones associated with formation of posterior cranial fontanel:** frontals and parieto-supraoccipital (state 0); exclusively frontals (state 1).

**26. Posterior cranial fontanel:** absent (state 0); present (state1).

**27. Size of posterior cranial fontanel (minimally connected):** reduced to a small opening (state 0); relatively narrow and long (state 1); wide and long (state 2); very wide and long (state 3).

**28. Epiphyseal bar:** conspicuous (state 0); indistinct (state 1).

**29. Size of epiphyseal bar:** transversely short and longitudinally wide (state 0); transversely elongate and longitudinally narrow (state 1).

**30. Medial groove of cranium:** present (state 0); absent (state 1).

**31. Form of medial groove of cranium:** shallow with margins not very conspicuous (state 0); deep with margins very conspicuous (state 1).

**32. Delimitation of medial groove (minimally connected):** delimited mainly by parieto-supraoccipital (state 0); delimited mainly by frontals (state 1); delimited exclusively by frontals (state 2).

**33. Bones forming cephalic shield:** smooth or grooved (state 0); granulated (state 2) smooth or grooved and granulated (state 1) .

**34. Fenestra delimited by parietosupraoccipital, pterotic, and sphenotic:** absent (state 0); present (state 1).

**35. Shape of extrascapular (minimally connected):** subrectangular (state 0); subquadrangular (state 1); subtriangular (state 2).

**36. Temporal fossa:** present (state 0); absent (state 1).

**37. Size of the temporal fossa*:*** very reduced (state 0); moderate to very large (state 1).

**38. Participation of extrascapular in delimitation of temporal fossa:** less than one fifth (state 0); more than three fifths (state 1).

**39. Epioccipital:** not exposed dorsally (state 0); dorsally visible (state 1).

**40. Epioccipital posterior process:** absent (state 0); present (state 1).

**41. Length of epioccipital posterior process:** short (state 0); very long (state 1).

**42. Relationship between epioccipital posterior process and crests associated with neural spine of fourth vertebra (minimally connected):** contacting a small narrow area of the diagonal crest (state 0); contacting both the diagonal and transversel crests (state 1); contacting a large area of diagonal crest (state 2).

**43. Relationship between epioccipital posterior process and medial crest associated with neuralspine of fourth vertebra:** not connected (state 0); connected (state 1).

**44. Shape of parieto-supraoccipital process (maximally connected):** base almost as narrow as posterior portion (state 0); base distinctly wider than posterior portion (state 1); base conspicuously narrower than posterior portion (state 2).

**45. Margin of pariet-osupraoccipital process contacting nuchal plate:** concave or notched (state 0); convex (state 1).

**46. Ventral crest of parieto-supraoccipital process:** weakly developed, restricted to base of process (state 0); well developed through entire extension of process (state 1).

**47. General shape of vomer (maximally connected):** diamond shaped (state 0); T-shaped (state 1); ovoid (state 2); narrow-shaped (state 3).

**48. Outline of vomer anterior margin:** weakly pronounced and serrated (state 0); very pronounced and acute (state 1).

**49. Vomer lateral processes:** absent (state 0); present (state 1).

**50. Length of vomer lateral processes:** long (state 0); short (state 1).

**51. Width of vomer lateral processes:** very wide (state 0); narrow (state 1).

**52. Width of anterior portion of posterior process of vomer:** wider than posterior portion (state 0); as narrow as distal portion (state 1).

**53. Tooth plates associated with vomer:** absent (state 0); present (state 1).

**54. Shape of vomerine tooth plates (maximally connected):** transversely elongate (state 0): rounded (state 1); butterfly-shaped (state 2); fused as a single large plate (state 3).

**55. Type of attachment between tooth plates and vomer (minimally connected):** tooth plates directly attached to vomer (state 0); tooth plates attached to vomer by ligaments (state 1); tooth plates entirely free (state 2).

**56. Accessory tooth plates:** absent (state 0); present (state 1).

**57. Number of accessory tooth plates (maximally connected):** one pair (state 0); two pairs (state 1); several small plates (state 2).

**58. Shape of accessory tooth plates (maximally connected):** small, transversely elongate, and narrow (state 0); small and rounded (state 1); large, oval to subtriangular (state 2); small, vertically oval (state 3); large, longitudinally elongate (state 4); moderately large, lateral and irregularly rounded (state 5).

**59. Shape of accessory tooth plates teeth:** needle-like (state 0); molariform (state 1).

**60. Location of accessory tooth plates (maximally connected):** apposed to lateral ethmoid (state 0); apposed to orbitosphenoid and metapterygoid (state 1); situated between premaxilla and lateral ethmoid (state 2).

**61. Relationship between lateral margins of orbitosphenoid:** uniformly parallel along their entire length (state 0); progressively diverging anteriorly (state 1).

**62. Lateral expansions of orbitosphenoid and pterosphenoid:** absent (state 0); present (state 1).

**63. Shape of orbitosphenoid and pterosphenoid lateral expansions (maximally connected):** two leaf-like short and wide processes (state 0); very narrow and long (state 1); slight projections with sinuous lateral face (state 2); slight projections with straight lateral faces (state 3).

**64. Parasphenoid in ventral view:** moderately wide (state 0); very wide (state 1).

**65. Size of optic foramen (minimally connected):** very large (state 0); moderately large (state 1); very reduced (state 2).

**66. Distance between optic foramen and trigeminofacialis foramen:** small, equal to width of trigemino-facialis foramen (state 0); large, about twice as large as trigemino-facialis foramen (state 1).

**67. Otic capsules:** well differentiated (state 0); weakly differentiated (state 1).

**68. Size of otic capsule (minimally connected):** very small, restricted to prootic (state 0); moderate, limited by prootic, pterotic and exoccipital (state 1); very large, limited by prootic, pterotic, and exoccipital (state 2).

**69. Enclosure of aortic canal:** absent (state 0); present (state 1).

**70. Position of anterior opening of aortic canal (minimally connected):** slightly anterior of subvertebral process and ventrally orientated (state 0); at base of subvertebral process and anteroventrally orientated (state 1); within base of subvertebral process and anteriorly orientated (state 2).

**71. Subvertebral process:** indistinct or weakly developed (state 0); well developed (state 1).

**72. Size of subvertebral process:** short and wide (state 0); long and narrow (state 1).

**73. Ventral tip of subvertebral process (minimally connected):** split (state 0); rounded (state 1); acute (state 2); spatulate (state 3).

**74. Anterior margin of subvertebral process:** smooth (state 0); keeled (state 1).

**75. Basioccipital lateral process:** absent (state 0); present (state 1).

**76. Basioccipital lateral process:** anterior and posterior portions equally developed (state 0); posterior portion extending further laterally than anterior (state 1).

**77. Length of basioccipital lateral process:** short (state 0); very long (state 1).

**78. Contact face for articulation of transcapular process with basioccipital:** small and columnar (state 0); large and depressed (state 1).

**79. Disposition of transcapular process in relation to body axis:** forming an acute angle (state 0); forming a right angle (state 1).

**80. Length and thickness of transcapular process:** long and thin (state 0); very short and thick (state 1).

**81. Shape of transcapular process:** cylindrical or columnar (state 0); depressed (state 1).

**82. Space between transcapular process and otic capsule (minimally connected):** very large (state 0); moderately large (state 1); very small (state 2).

**83. Posterior process of exoccipital:** absent (state 0); present (state 1).

**84. Bony crest of exoccipital:** well developed and tall (state 0); shallow and inconspicuous (state 1).

**85. Orientation of exoccipital bony crest (maximally connected):** perpendicular to vertebral column and ventrolaterally directed (state 0); perpendicular to vertebral column and posteriorly directed (state 1); parallel to vertebral column and mesially folded (state 2); parallel to vertebral column and posteriorly directed (state 3).

**86. Connection between posterior process of exoccipital and Müllerian ramus:** by ligaments (state 0); by suture (state 1).

**87. Relationship between exoccipital posterior process and Müllerian ramus:** posterior process not supporting Müllerian ramus (state 0); posterior process supporting Müllerian ramus (state 1).

**89. Shape of anterior infraorbital:** straight or slightly curved (state 0); conspicuously curved (state 1).

**90. Shape of posterior infraorbital (maximally connected):** C-shaped (state 0); S-shaped (state 1); L-shaped (state 2).

**91. Number of branches in lachrymal-antorbital anterior part (maximally connected):** two anterior branches and one lateral (state 0); three anterior branches and one lateral (state 1); two anterior branches and one mesial (state 2); three anterior branches (state 3)

**92. Posterior branch of lachrymal-antorbital:** short and little differentiated (state 0); long and conspicuous (state 1).

**93. Width of lachrymal-antorbital:** very wide (state 0); narrow (state 1).

**94. Shape of maxilla (maximally connected):** laminar and leaf shaped (state 0); cylindrical, moderately long, and distally acute (state 1); cylindrical, very long, and distally acute (state 2); rudimentary and weakly differentiated (state 3).

**95. Outline of maxilla (maximally connected):** mesial and lateral margins parallel in proximal two thirds, converging in distal one third, distal margin truncate (state 0); wide for proximal two thirds with edges parallel, narrow distally, and thin and acute posteriorly (state 1); maxilla gradually narrow distally, but lateral and posterior margins rounded (state 2); lateral and mesial margins considerably closer to each other proximally, distally narrow, and pointed (state 3).

**96. Size of maxillary condyles (minimally connected):** rudimentary (state 0); moderately large (state 1); large (state 2); very large (state 3).

**97. General aspect of autopalatine (maximally connected):** cylindrical, long, and thin (state 0); conical, short, and robust (state 1); cylindrical, very short, and robust (state 2); depressed and mesially angled in its anterior third (state 3).

**98. Autopalatine anterior portion:** depressed (state 0); cylindrical (state 1).

**99. Autopalatine posterior portion (maximally connected):** slightly compressed (state 0); conspicuously compressed (state 1); conspicuously depressed (state 2).

**100. Autopalatine orientation:** parallel in relation to main body axis (state 0); perpendicular to the main body axis (state 1).

**101. Shape of autopalatine at its articulation with lateral ethmoid (maximally connected):** slightly compressed (state 0); very compressed (state 1); depressed (state 2).

**102. Size of articulation of autopalatine with lateral ethmoid:** very large (state 0); moderately large (state 1).

**103. Orientation of articulation of autopalatine with lateral ethmoid (minimally connected):** mesially orientated (state 0); mesoposteriorly orientated (state 1); posteriorly orientated (state 2).

**104. Position of articulation of the autopalatine with lateral ethmoid (minimally connected):** slightly displaced to anterior portion of bone (state 0); in the middle of bone (state 1); slightly displaced to posterior portion of bone (state 2).

**105. Autopalatine ventral process:** absent (state 0); present and very conspicuous (state 1).

**106. Length of anterior cartilage of autopalatine (minimally connected):** very short, less than one third as long as bone itself (state 0); moderately long, one third to one fifth as long as bone itself state 1); very long, about as long as bone itself (state 2).

**107. Size of autopalatine posterior cartilage (minimally connected):** as long as anterior cartilage (state 0); much smaller than anterior cartilage (state 1); reduced to a small dot (state 2).

**108. Ventral process at symphysis of dentary (minimallyconnected):** absent (state 0); short and inconspicuous (state 1); long and very conspicuous (state 2).

**109. Distribution of teeth on the dentary (maximally connected):** restricted to mesial two thirds (state 0); restricted to mesial three quarters (state 1); along entire bone (state 2).

**110. Types of teeth on dentary (maximally connected):** teeth acicular (state 0); teeth acicular and molariform (state 1): teeth spatulate or cuspidate (state 2).

**111. Anterodorsal process of anguloarticular:** absent (state 0); present (state 1).

**112. Presence of a rod-like structure connecting jaw coronoid process and base of maxillary barbel:** absent (state 0); present (state 1).

**113. Relationship between length and width of premaxilla (maximally connected):** premaxilla wide and short (state 0); premaxilla wide and moderately long (state 1); premaxilla narrow and very long (state 2); premaxilla very wide and short (state 3).

**114. Length variation of lateral and mesial portions of premaxilla:** lateral and mesial portions about equally developed (state 0); lateral and mesial portions with different sizes (state 1).

**115. Anterior margin of premaxilla:** entire (state 0); fringed (state 1).

**116. Shape of lateral margin of premaxilla (minimally connected):** straight or slightly convex (state 0); with a slight concavity (state 1); with a very conspicuous concavity (state 2).

**117. Dorsal crest on the premaxilla delimiting a contact area with mesethmoid:** dorsal crest beginning near or exactly at lateral end of premaxilla anterior margin (state 0); beginning between lateral one third or half of anterior margin (state 1).

**118. Dorsal crest of premaxilla:** present (state 0); absent (state 1).

**119. Teeth in the premaxilla:** acute (state 0); cuspidate (state 1).

**120. Shape of anteroventral portion of opercle (maximally connected):** subtrapezoid, very long (state 0); subtrapezoid, moderately long (state 1); subtrapezoid, very short (state 2); subrectangular (state 3); subtriangular (state 4).

**121. Shape of anteroventral margin of opercle:** slightly convex (state 0); concave or almost straight (state 1).

**122. Opercle posterior portion:** not well developed posteriorly (state 0); well-developed sterodorsally (state 1).

**123. Shape of posterior margin of interopercle:** slightly curved (state 0); straight and inclined (state 1).

**124. Contact area between posterior face of interopercle and ventral margin of opercle:** half or less of interopercle posterior part contacting ventral margin of opercle (state 0); more than half of interopercle posterior part contacting ventral margin of opercle (state 1).

**125. Anterior portion of interopercle:** columnar and truncate (state 0); compressed and bifurcate (state 1).

**126. Interopercle anterior portion (minimally connected):** inconspicuous (state 0); conspicuously narrow (state 1); thin and acute (state 2).

**127. General shape of interopercle (maximally connected):** subtriangular (state 0); rectangular (state 1); subrectangular (state 2).

**128. Shape of metapterygoid in perpendicular section (maximally connected):** twice as deep as long (state 0); as deep as long (state 1); one and a half times longer than deep (state 2); subtriangular (state 3); three times longer than deep (state 4).

**129. Articulation between metapterygoid and quadrate:** by complete interdigitated suture (state 0); by interdigitated suture in a small part of contact and by overlapping in the remaining contact area (state).

**130. Anterior process of metapterygoid:** present (state 0); absent (state 1).

**131. Shape of metapterygoid anterior process (minimally connected):** acute (state 0); truncate (state 1); rounded (state 2).

**132. Size of metapterygoid anterior process:** small to moderate (state 0); very large (state 1).

**133. Dorsal crest of hyomandibular:** absent (state 0); present (state 1).

**134. Shape of dorsal crest of hyomandibular:** long and low (state 0); short and high (state 1).

**135. Ventral crest of hyomandibular:** present (state 0); absent (state 1).

**136. Process of hyomandibular for insertion of the adductor mandibulae:** inconspicuous or absent (state 0); very conspicuous (state 1).

**137. Contact face between metapterygoid and hyomandibular:** moderately long (state 0); very long (state 1).

**138. Shape of sesamoid bone I (maximally connected):** short and triangular (state 0); very long and subtriangular (state 1); irregularly shaped (state 2); very long and subrectangular (state 3).

**139. Shape of sesamoid bone II:** half-moon shaped (state 0); irregularly elongate (state 1).

**140. Shape of first external branchiostegal ray***:* proximally narrow and distally broad (state 0); as broad proximally as distally (state 1).

**141. Second external branchiostegal ray:** width less than half that of first ray (state 0); almost as wide as first ray (state 1).

**142. The distal portion of third external branchiostegal ray:** acute (state 0); spatulate (state 1).

**143. Anterior portion of anterior ceratohyal:** columnar to cylindrical (state 0); compressed (state 1).

**144. Posterior portion of anterior ceratohyal:** compressed and moderately thick (state 0); columnar and very thick (state 1).

**145. Thickness of anterior portion of anterior ceratohyal:** thin to moderately thick (state 0); very thick (state 1).

**146. Size of posterior ceratohyal (minimally connected):** short (state 0); long (state 1); very long (state 2).

**147. Profile of anterior margin of urohyal:** notched (state 0); not notched (state 1).

**148. Posterolateral processes of urohyal:** absent (state 0); present (state 1).

**149. Dorsal crest of urohyal:** not projected anteriorly (state 0); projected anteriorly (state 1).

**150. Length of urohyal (minimally connected):** short (state 0); long (state1); very long (state 2).

**151. Posterior end of urohyal:** acute (state 0); bifurcate (state 1).

**152. Bony blade connecting posterolateral processes of urohyal:** present (state 0); absent (state 1).

**153. Size of posterolateral processes of urohyal:** short (state 0); long (state 1).

**154. Orientation of posterolateral processes of urohyal:** lateroposteriorly orientated, forming an angle always larger than 70° (state 0); posteriorly orientated, forming an angle smaller than 60° (state 1).

**155. Relationship between length of urohyal and length of posterolateral processes (maximally connected):** posterolateral processes almost as long as distal portion of bone (state 0); posterolateral processes as long as or longer than distal portion of bone (state 1); posterolateral processes two thirds as long as distal portion of bone (state 2); posterolateral processes more than half as long as distal portion of bone (state 3); posterolateral processes one third as long as distal portion of bone (state 4).

**156. Shape of posterior portion of urohyal:** posteriorly narrow and acute (state 0); as wide distally as proximally (state 1).

**157. Number of components of basibranchial series:** two (state 0); one (state 1).

**158. Anterior portion of second basibranchial:** not expanded (state 0); very expanded (state 1).

**159. Shape of second basibranchial:** spindle-shaped (state 0); mushroom-shaped (state 1).

**160. Relationship between length and width of posterior portion of second basibranchial (maximally connected):** long and wide (state 0); very long and narrow (state 1); long and wide (state 2); short and wide (state 3); short and narrow (state 4).

**161. Shape of third basibranchial:** hourglass-shaped (state 0); chalice-shaped (state 1).

**162. Length and width of third basibranchial (maximally connected):** very short and wide (state 0); moderately long and wide (state 1); long and narrow (state 2); very long and narrow (state 3).

**163. Median constriction of third basibranchial:** approximately at the middle of the bone (state 0); displaced to posterior quarter (state 1).

**164. Shape of first hypobranchial:** shell-like (state 0); club-shaped (state 1).

**165. First hypobranchial:** moderately elongate transversely, its mesial face weakly developed and rounded (state 0); very elongate transversely, its mesial face well developed and acute (state 1).

**166. Anterior process of first hypobranchial:** inconspicuous (state 0); very conspicuous (state 1).

**167. Position of anterior process of first hypobranchial:** slightly displaced to lateral part of the bone (state 0); exactly in the middle of the bone (state 1).

**168. Shape of second hypobranchial:** shell-like (state 0); club-shaped (state 1).

**169. Shape of second hypobranchial:** slightly elongatebtransversely, its mesial face rounded (state 0); very elongate transversely, its mesial face acute (state 1).

**170. Contact face between first epibranchial and first pharyngobranchial:** inconspicuous (state 0); very conspicuous (state 1).

**171. Shapes of first and second epibranchials:** curved at mesial third (state 0); straight along their entire extension (state 1).

**172. Disposition of first and second epibranchials:** first epibranchial parallel to second (state 0); first epibranchial overlaying second (state 1).

**173. Mesial portion of first epibranchial (maximally connected):** tubular and slightly depressed (state0); large and depressed (state 1); very large and depressed (state 2).

**174. Posterior margin of first epibranchial:** straight (state 0); mesial one third with a prominent process (state 1).

**175. Size of uncinate process of third epibranchial:** equal or smaller than mesial portion of thirdepibranchial delimited by uncinate process (state 0); much longer and widerthan mesial portion of third epibranchial delimitedby uncinate process (state 1).

**176. Distal portion of uncinate process of third epibranchial:** acute (state 0); truncate (state 1).

**177. Shape of uncinate process of third epibranchial (minimally connected):** mesially curved and length variable (state 0); straight and elongate (state 1); laterally curved and short (state 2).

**178. Margin of lateral uncinate process of third epibranchial:** straight (state 0) notched (state 1).

**179. Mesial quarter of fourth epibranchial:** robust, almost as wide as long (state 0); thin, its width about twice its length (state 1).

**180. Posterior margins of fourth epibranchial:** conspicuously convex, half as wide as long (state 0); slightly convex, one quarter as wide as long (state 1).

**181. Anterior portion of proximal cartilage of fourth epibranchial:** as large as posterior portion (state 0); about twice narrower than posterior portion (state 1).

**182. First pharyngobranchial:** present (state 0); absent (state 1).

**183. Shape of first pharyngobranchial:** long and narrow (state 0); large and depressed (state 1).

**184. Location of first pharyngobranchial in relation to first epibranchial:** on mesial end of first epibranchial (state 0); on mesial two thirds of first epibranchial (state 1).

**185. Shape of third pharyngobranchial:** funnel-like (state 0); angled in form of boomerang (state 1).

**186. Lateral margin of third pharyngobranchial:** weakly developed and rounded (state 0); well developed and acute (state 1).

**187. Space for insertion of teeth on fifth ceratobranchial (minimally connected):** very large (state 0); moderate (state 1); very small (state 2).

**188. Shape of upper (pharyngeal) tooth plate (minimally connected):** round, as wide as long (state 0); oval-shaped, its width twice its length (state 1); long and narrow, its width three times its length (state 2); very long and narrow, its width more than four times its length (state 3).

**189. Dorsal processes of upper (pharyngeal) tooth plate:** free (state 0); connected by bony blade (state 1).

**190. Length of dorsal processes of upper (pharyngeal) tooth plate (minimally connected):** very short or absent (state 0); long (state 1); very long (state 2).

**191. Diagonal crest associated with posterior branch of parapophysis of complex vertebra:** indistinct (state 0); well developed (state 1).

**192. Extension of diagonal crest associated with posterior branch of parapophysis of complex vertebra:** short, reaching transverse crest (state 0); long, reaching base of Müller’s ramus (state 1).

**193. Transverse crest associated with neural spine of fourth vertebra:** low (state 0); very high (state1).

**194. Median crest associated with neural spine of third vertebra***:* low or absent (state 0); very high (state 1).

**195. Median crest associated with neural spine of fourth vertebra:** low or absent (state 0); very high (state 1).

**196. Size of the opening delimited by epioccipital posterior process and the crests of sustentaculum of Weberian apparatus (minimally connected):** very large (state 0); moderate (state 1); very reduced (state 2).

**197. Accessory crest connecting transverse and median crests associated with neural spine of fourth vertebra:** absent (state 0); present (state 1).

**198. Opening delimited by posterior process of epioccipital and crests associated with sustentaculum of Weberian apparatus:** absent (state 0); present (state 1).

**199. Superficial ventral ossification of the Weberian apparatus:** not or only partially covering the aortic canal (state 0); entirely covering the aortic canal (state 1).

**200. Location of cardinal veins in relation to aortic canal:** above level of aortic canal (state 0); at same level of aortic canal (state 1).

**201. General aspect of superficial ventral ossification:** keeled (state 0); regularly arched (state 1).

**202. Ventral surfaces of parapophyses of fifth and sixth vertebrae:** straight (state 0); conspicuously concave (state 1).

**203. Number of ribs:** 14 or fewer (state 0); 15 or more (state 1).

**204. First vertebra free from ventral superficial ossification (minimally connected):** eighth vertebra (state 0); seventh vertebra (state 1); sixth vertebra (state 2); fifth vertebra (state 3).

**205. Number of precaudal vertebrae:** 18 or fewer (state 0); 19 or more (state 1).

**206. Number of caudal vertebrae:** 39 or more (state 0); 38 or fewer (state 1).

**207. Anterior and middle nuchal plates:** distinct (state 0); indistinct (state1).

**208. Shape of complex formed by anterior and median nuchal plates (maximally connected):** half-moon shaped (state 0); shield-like (state 1); butterfly shaped (state 2).

**209. Nuchal plate anterior margin (maximally connected):** convex (state 0); slightly concave (state 1); conspicuously notched (state 2).

**210. Contact between nuchal plate and parieto-supraoccipital (maximally connected):** contact made through a deeply notched articulation (state 0); nuchal plate and parietosupraoccipital contacting one another through a convex-concave articulation (state 1); nuchal plate overlaying parieto-supraoccipital (state 2).

**211. Dorsal-fin spine:** without filament (state 0): prolonged into a filament (state 1).

**212. Length of adipose-fin base (minimally connected):** very long (state 0); long (state 1); moderately long (state 2); very short (state 3)

**213. Position of adipose-fin origin (minimally connected):** anterior to anal-fin origin (state 0); vertically above anterior half of anal fin (state 1); vertically above posterior half of anal fin (state 2).

**214. Length of posterior process of cleithrum (minimally connected):** very short (state 0); moderately long (state 1); very long (state 2).

**215. Position of the second dorsal cleithral process:** on upper portion of cleithrum (state 0); on lower portion of cleithrum (state 1).

**216. Orientation of the second dorsal cleithral process:** posteriorly directed and parallel to posterior process (state 0); dorsally directed and parallel to first dorsal process (state 1).

**217. Cleithrum lateral face:** moderately wide (state 0); very narrow (state 1).

**218. Relationship between posterior process of cleithrum and cleithrum second dorsal process:** free (state 0); connected by a bony blade (state 1).

**219. Mesocoracoid loop:** present (state 0); absent (state 1).

**220. Pelvic splint:** absent (state 0); present (state 1).

**221. Distance between nostrils:** anterior nostril distant from posterior (state 0); anterior and posterior nostrils close together (state 1).

**222. Groove connecting posterior nostrils:** absent (state 0); present (state 1).

**223. Maxillary barbels:** present (state 0); absent (state 1).

**224. Maxillary barbel:** cylindrical (state 0); compressed (state 1).

**225. Mental barbels:** present (state 0); absent (state 1).

**226. Number of mental barbels:** two pairs (state 0); one pair (state 1).

**227. Swimbladder:** with a single chamber (state 0); with three chambers (state 1).

**228. Shape of lateral line at caudal region:** not bifurcated (state 0); bifurcated (state 1).

**229. Origin of protractor muscle of parapophysis of fourth vertebra:** from ventral surface of parieto-supraoccipital process and posterior process of epioccipital (state 0); exclusively from ventral surface of posterior process of epioccipital (state 1).

**230. Contact between protractor muscle of parapophysis of fourth vertebra and posterior process of epioccipital:** absent (state 0); present (state 1).
