# Supplementary material for: A new genus and species of marine catfishes (Siluriformes; Ariidae) from the upper Eocene Birket Qarun Formation, Wadi El-Hitan, Egypt
Source: PLoS One. 2017 Mar 1;12(3):e0172409. doi: 10.1371/journal.pone.0172409 (PMC5332075; doi:10.1371/journal.pone.0172409)
Supplement: S2 Appendix — Character matrix of 230 characters for the Ariidae species and outgroups. (PDF) [file pone.0172409.s002.pdf]

```
#NEXUS

BEGIN DATA;
      DIMENSIONS  NTAX=94 NCHAR=230;
      FORMAT DATATYPE = STANDARD GAP = - MISSING = ? SYMBOLS = "  0 1 2 3 4 5 6
7 8 9";
      MATRIX
      Diplomystidae                000-001000---1---000-000011001--000---00----
01000--01100----10-1--000-0---0--000000----0-----0--00-
00?010010?0000000?10000000002-00110001--010000010000----000-21?-
0000000000001000000000-11--0-010-0000-003010-0-000100000110001-0---
      Cetopsidae                   000-00-0010--1---000-000011001--00----00---
1011?10001-00----00-0--000-0---0--000000----2-----03-
00200?010010?0000000?10?000?00020?--11001--000100010000----000-20?-
0000000000000?00000000-21000-010-0000-00201--000-------010000000?--
      Ictaluridae                  120-0000010-12---000000011001---00---00---
1011010000--0----00-000000-0---0--000000----1-
001100101000?010010?0000000?100000000?000110101--100000000000----000-21?-
0000000000000000001--0-11000-010-0000-002010-000211010001000000---
      Cranoglanididae              000-00?0011112---0000010-100001020-1--00---
1011110000--0----00-000000-0---0--000000----2-
001100101000?010010?0000000?10000000102000010-101--000000000000----000-2??-
0000000000001000001--0-11000-010-0000-0?2??0-0002120100010000000?--
      Doradidae                    000-00000----1---00000?0-0-1-1--20-1--110-
01001010000--0----00-000000-0---100010000----
300011001010000?010010?0000000?100000010001--11101--010000000000----0?0-???-
0100010000000000001--0-11000-0?0-0000-001010-0-000201000?0000000?-0
      Mochokidae                   0?0-0000010--1---000000112001--20-1--00---
1001010000--0----00-000000-0---10001000-----
30001100101000?010010?0000000?10000000101?1--1110?--000000000010----000-???-
010001000-000000001--0-11000-010-0000-001010-0-000----1010000000--0
      Pangasidae                   020-0000010-1310100001100011001--000---010-
0101101000100100000000-000000-0---0--000001--002-
10110210100?010010?0001000?104000000?0001110000-000000000000----000-?1?-
0000000000000100000000-11000-010-0000-001010-000321100001000000101-0
      Schilbeidae                  020-00010---131010000111011001--000---010-
0101101000100100000010000000-0---0--000001--002-
10110210100?010011?0002000?10400000?20001100000-000000000010----000-2??-
0000000000001100000000-11000-010-0000-001010-0-03210000010000000?--
      Bagridae                     100-00000---12---000000011001--200---00---
0001010001000----010000000-0---0--000000----1-
001100101000?010010?0000000?10000000?020000101l?0-100000000010----000-21?-
0000000000001000000000-11000-010-0000-001010-00000?000001000000---
      Horabagridae                 010-000001001 2---0000010-0---1---00---00---
1011010001000----00-0-0010-0---0--000000----2-
00110200100?010010?0001000?10?0000001200001 1010--000000100010----000-???-
00000000010100000000000-11000-011-0000-001010-00032100000100000000--
      Pimelodidae                  100-0000010-12---0000000-0-001--200---00---
1011???001000----0100?0000-0---0--00000-----
30001100101000?010010?0000000?10401000101000000-100--010000000010----000-2??-
000000000000?000001--0-11000-011-0000-002010-00000----10?0000000?--
      Claroteidae                  000-0000010-12---000010110001--000---00---
101111000100100000100000000-0---0--000001--002-
10110210100?010111?0010000?1040100000?0001???1?0-0000001011000000000-2??-
0?000?0000000000000000-11000-010-0000-002010-000000010000000000000?--
```

```
      Austroglanididae            000-0000010-12---00000-0011001--000?-?00---
1011110000--0----00-0?0000-0---0--000000----2-10110-
101000?010110?0000000?1040000000?000111001--01000000100----000-
?0?00?000?0000100000001--0-11000-010-0000-001010-000002000001000000?--
      Ancharidae                  010-0000010-12---0000010011000100000l000---
1011010000--0----00-?20000-0---0--000000----2-
00110200100?010111?0010001?1040100000000000110100-0000000011000000000-2??-
00000000000000100001--0-11000-010--000-0020110000001010010000000?-0
      Arius_arius                 100-
00000112030010001001011010102000100112010011l0000--
104110120110211100011000001101013010100210100110201111001100110010101000100000110
10101000000010100102001l211-
000000100000110101001111101110002011000?111001121101001010000000111
      Arius_caelatus              110-
0000011003001000l1001?11000102000100112010011l0000--
10200010011021110001100000110101301010021010?10201111001100110010?01000l0000110
10100000000010100102001l211-
000000?000001101010011111011100020110000011100112110100101000000111
      Arius_dispar                1?0-
000001120300100010010110001020001001120100111l0000--
102110120110211100011000001101013010100210100110201111001100110010101000100000110
101010000000101001020011211-0000000000001101010011-
110111000201100000111001021101001010000000111
      Arius_gagora                100-
000001120300100010010110101020001001120100111l0000--
104110120110211100011010001101013010100210100110201111001100110010101000100000110
101010000000101001020011411-000000100000110101001l-
11011?00020110000011100102110100101000000111
      Arius_maculatus             110-
000001120300100010010110001020001001120100111l0000--
102000120110211100011000001101013010100210100110201111001100110010101000100000110
10?0?00000001010010?0011411-000000100000110101001l-
110111000201100000111001121101001010000000111
      Arius_madagascariensis      100-
000001120300100010010110001020001001120100111l0000--
104110100110211100011010001101013010100210100110201111001100110010101000100000110
101010000000101001020011211-
000000000000110101001111101110002011000001110011211010010100000000111
      Arius_manillensis         100-
000001120300100010010110001020001001120100111l0000--
104110120110211100011010001101013010100210100110201111001100110010101000100000110
101010000000101001020011211-
000000000000110101001111101110002011000001110010211010010100000000111
      Potamosilurus_velutinus     110-
00000111030010001011011000102000100112010010100000--0----
010011021110001100000111001301010021010001020111100110011001010100010000l101000-
000000010100100001120100000000100000110101001111101110002011000001110010211010010
1000000011
      Aspistor_luniscutis         110-000001010301100010010l2011--
2010101110011111100013010411011111021111201101l001003--
30101002101000102011110011001000101010001000011010201000000011100102101l411-
000000000000110101001101101100100111000011112120111000010100000000l
      Aspistor_quadriscutis       110-000001010301100010010l2011--
2010101110011111100013010411011111021111201101l001003--
```

```
301010021010001020111100110010001010100010000110102010000000111001021011411-
0000000000001101010011011011001001110000111121201110000101000000001
      Bagre_bagre                 020-
01010110031010001111110000100000100110010110100010010000011010021010101101000110
1003122101-2110000000101110010001100001000000000010-
0101000000010101101010010201100000000100011010100110110110111001?0001?1000001321
1000101001010101
      Bagre_marinus               020-
01010110031010001111110000100000100110010110100010010000011010021010101101000110
1003122101-2110000000101110010001100001000000000010-
01010000000101011010100102011000000001000110101001101101101110011000101010001321
1000101001010101
      Bagre_panamensis            020-
00010100031010001111110000000000000110010110100010010000011010021010101101000110
1003122101-2110000000101110010001100000000000000010-
01000000000101011011100102011000000001000110101001101101101110011000101010000321
1000101001010101
      Bagre_pinnimaculatus        020-
01010100031010001111110000000000000110010110100010010000011010021010101101000110
1003122101-2110000000101110010001100001000000000010-
0101000000010101101010010201100000000100011010100110110110111001?000101010001321
1000101001010101
      Batrachocephalus_mino       -20-000000--130001201010-0-1-
0102000100111010010100000--1031200-0110211112011000002101013012103-
03021212202111201100110130100000301--11011?-01001011101000020010212-
000000000000110111001102101111001011000000110010211000010101-000101
      Brustiarius_nox             120-
00000100031010001011110000101000000112010010101012010100010111021010001100000111
000301010031010001010111100101010001010000010000100100000000001010100110001020100
0000010000011010100111110111000201100000011001032100001010000000011
      Brustiarius_solidus         120-
00000100031010001011110000101000000112010010101012010100010111021010001100000111
000301010031010001010111100101010001010000010000100100000000001010100110001020100
0000010000011010100111110111000201100000111001032100001010000000011
      Cathorops_agassizii
10101112011103212000100111000010202001011101011010100--
10310011011121211311111101210200301010021010001020112110120010002111110020020111
101011010-
01111000020011201001100100000011010100110210110111001111001011001031001101010000
00001
      Cathorops_spixii
10101112011103212000100111000010202001011101011010100--
10310011011121211311111101210200301010021010001020112110120010002111110020020111
101011010-
01111000020011201001100100000011010100110210110111001111001011001031001101010000
00001
      Cathorops_arenatus
10101112011103212000100111000010202001011101011010100--
10310011011121211311111101210200301010021010001020112110120010002111110020020111
101011010-
01111000020011201001100100000011010100110210110111001111001011001031001101010000
00001
      Cathorops_dasycephalus      100-
01100111032120001001011000102020000111010110101011010300010011121211311111101210
200301010021010001020111100120010002111110010020111101011010-
```

011110000200112010000000000000110101011101101101110011010010110010311010010100000
00001
Cathorops_fuerthii
1010111201110321200010011100001020200101110101101010O--
10310011011121211311111101210200301010021010001020112110120010002111110020020111
101011010-
01111000020011201001100100000011010100110210110111001111001011001031001101010000
00001
Cathorops_hypophthalmus
10101112011103212000100111000010202001011101011010100--
10310011011121211311111101210200301010021010001020112110120010002111110020020111
101011010-
01111000020011201001100100000011010100110210110111001111001011001031001101010000
00001
Cathorops_multiradiatus
10101112011103212000100111000010202001011101011010100--
10310011011121211311111101210200301010021010001020112110120010002111110020020111
101011010-
01111000020011201001100100000011010100110210110111001111001011001031001101010000
00001
Cathorops_tuyra
10101112011103212000100111000010202001011101011010100--
10310011011121211311111101210200301010021010001020112110120010002111110020020111
101011010-
01111000020011201001100100000011010100110210110111001111001011001031001101010000
00001
Cephalocassis_borneensis 100-01100101032120001000013011--21100-
011101011110100--0----00-
02102111130110000021010030101002101000102011100012001000201010102000010110101100
00001110000200102010011001000000010111001101101100100111010010110010110011010100
0000101
Cephalocassis_melanochir 100-01100101032120001000013011--
211010011000011110100--1030000-021021111300--
00002101003010100210100010201110001200100010101010200001011010110000001010010000
1020100110010000000101110011011011001001110000101100101100110101000000001
Cinetodus_froggatti 000-0000011103101000101101-001--
202010111001011110000--10-
00010011021111201000110210100301010021010001020111100120010000010100020010100101
010000000101000020010211-000000000000101010-
11010001011100110010101100101120000101000000001
Cochlefelis_danielsi 120-
00000100031000001011011000001010100112010010101011010000011111021010001100000111
00030101003101000101011110010001100401000001001010010000000001010100110001020200
0000000000011000100110110111000201100010101001021100001010000000011
Cochlefelis_spatula 120-
00000101031000001011011000001010100112010010101011010000011111021010001100000111
00030101003101000101011110010001100401000001001010010000000001010100110001020200
0000000000011000100110110111000201100010101001021100001010000000011
Doiichthys_novaeguineae 020-011001020311201?-000013011--
201010011100001010100--1030000-
0210211113011010001101001??0?0021011001000111100130010001010101000000010010100000
002010210104001110300110010102000201110000-
22121000000110000101100102?00110101000000001
Galeichthys_ater 010-00000100031010001000110000000000000010-
000010100010010000010010020-10000--

```
0100111000301000011010000010001101100010000000000000000000-
0100001000000110000000010002000000000011000100011--1001010-000--000-001010000011-
--011010000000-0
      Galeichthys_feliceps          010-00000100031010001000110000000000000010-
000010100010010000010010020-10000--
0100111000301000011010000010001101100010000000000000000000-
0100001000000110000000010002000000000011000100011--1001010-000--000-001010000011-
--011010000000-0
      Genidens_barbus               110-
00000111030010001011110000102000000112010010101011112?0011001102101000110000011l
00130101000101000102011100011001000100010001000010010101000000001010000200103010 0
00000000000110101001101101110002011000101110010211010010100000001l
      Genidens_genidens             110-
000001110300100010111100001020000001120100101010 0--
12?00110011021010001100000111001301010001010001020111000110010001000100010000100
10101000000010100002001030100000000000000110101001101101110002011000101110010211 0
10010100000011
      Genidens_machadoi             110-
000001110300100010111100001020000001120100101010111102001100110210100011000001 11
001301010001010001020111000110010001000100010000100101010000000010100002001030100
00000000000110101001101101110002011000101110010211010010100000001 1
      Hemiarius_stormii             110-0000010103212000100101 2011--
2010100110010110101011010300011111021111301100000110100301010021010001020111100 1
100110010101000100001101010100000000101001000011211-
00000000000001010100110110110111001100001011001011001101010000000001
      Hemiarius_sumatranus          110-0000010103212000100101 2011--
2010100110010110101011010300011111021111301100000110100301010021010001020111100 1
100110010101000100001101010100000000101001000011211-
0000000000001101010011011011011100110000101100101100110101000000?01
      Ketengus_typus                0011111000---0---
1001010011000102000100111010000 0--00--0----00-011021111201100000210101301310 3-
03021212202112201 1001-1140000000301--11111?-01000000111000020 1------1---1-
001000110111001 0-21011-10010110000101100102110100101000000101
      Nedystoma_dayi                110-01100101031120001000013011--
20101001110001101010 0--0----00-
021021111301101000110100301010021011001020111100110110000010101010000110101011 10
00001010010200111030011001100000020111001112312101100011000010110010210011010100
0000001
      Nemapteryx_armiger            110-
011201010311200010000130101020101001110101101000 11010-0000-
021021111301101100210100301010021010001010111100110011001010100010000100101010 00
00001010010200112010000000000000110101001101101101100011000010110010210011010100
0000001
      Neoarius_graeffei             110-
00000111031010001011011000101010100112010010101011010000010011021010001100000111
00130101003101000101011110011001000101000001000010010000000000001010011000102010 0
00100000000110101001111101110002011000101110010211000010100000001 1
      Neoarius_midgleyi             110-
00000111031010001011011000101010100112010010101011010000010011021010001100000111
00130101003101000101011110011001000101000001000010010000000000001010011000102010 0
00100000000110101001111101110002011000101110010211000010100000001 1
      Netuma_bilineatus             110-0000011113000000101111000010 20100-
011201001010101101120001001102101000110000011100130101002101000102011110010001 10
010101000100001-01000000000000101001100011311-
00010000000011010100111110111000201100010111001032100001010000000111
```

```
      Netuma_thalassinus           -10-
000001111300000001011110000102010100112010010101011011200010011021010001100000111
0013010100010100010201111001000110010101000100001001000000000000101001020011311-
0001000000001101010011111011100020110001011100103210000101000000111
      Notarius_grandicassis        -10-00000101030010001011011011--
20101001100200111010110102000111110211112011011001003--
301010021010001020111100110010001010000010000110102000000000011100100001120100000
000000001101010011011011000001110001011100101110000101000000001
      Notarius_lentiginosus        110-00000101030010001011011011--
20101001100100111010110102000111110211112011011001003--
301010021010001020111100110010001010000010000110102000000000011100100001120100000
000000001101010011011011000001110001011100101110000101000000001
      Notarius_planiceps           110-
0000010103001000101101101000201010011001001010001101020001011102111120110110011-
-
003010100210100010201111001100100010100000100001101020100000000101001000011201000
000000000011010100110110110101?111000?0111001011100001010000000?1
      Notarius_troschelii          110-000001?1030010001011011011--
20101001100100111010110102000111110211112011011001003--
30101002101000102011110011001000010?0000010000110102000000000011100100001120100000
000000001101010011011011000001110001011100201110000101000000001
      Osteogeneiosus_militaris     -00-000000--
13002001000111100010200010011101001110010--1041100-
0110211112011000002101013011102-32100011200211001100100010101000401--
1101020000000000100000020011201000000001000001101010011011011-
100201100000111001021101001010001-0111
      Pachyula_crassilabris        000-00000111031010001011011001--
20?010111101011110000-?0----
01001102111120100011021010030101002101000102011110011001000201010002001010010101
0000000101000020010211-
000000000100101010111011011011100110010101100101120000101000000001
      Potamarius_izabalensis       100-
0000011203001000100111100010101010011001001110100--0----00-
00002101000110000011101-
30101003101000102011110012001000101010012001010010101000000010101101001020200000
000000001101010011011011000000110000011100102110100101000000001
      Potamarius_grandoculis       100-
0000011203001000100111100010100010011201001110100--10-0-00-
00002101000110000011100130101003101000102011110012001000101010012001010010101000
0000101011010010202000000000000001101010010-
11011100020110000011100102110100101000000011
      Sciades_assimilis            110-000001111300000001011-0-1-0112011--
011201001010001101020001001101100-0-110000001101-
3010100210100010201111001100100010001000100001001010100000000101001000011211-
0000000000001101010011011011100020110001011100102110000101000000011
      Sciades_bonillai             110-000001111300000001011-0-1-0112011--
011201001010001101020001001101100-0-110000001101-
3010100210100010201111001100100010001000100001001000100000000101001000011211-
0000000000001101010011011011100020110001011100102110000101000000011
      Sciades_couma                120-000001111300000001011-0-1-0022011--
011201001010001101020001011101100-0-110000001101-
3010100210110011201111001100200010101000100001001031100000000101001000011211-
0000000000001101010011011011100020110001011100102110000101100000111
      Sciades_felis                110-000001111300000001011-0-1-0112011--
011201001010001101020001001101100-0-110000001101-
```

```
301010021010001020111100110010001010100010000100100010000000101001000011211-
0000000000001101010011011011100020110001011100102110000101000000011
      Sciades_guatemalensis         120-00000111130000001011-0-1-0022011--
011201001010001101000001001101100-0-110000001101-
301010021011001120111100110010001000100010000100101010000000101001000011201000000
000000001101010011011011100020110001011100102110000101000000011
      Sciades_herzbergii            120-00000111130000001011-0-1-0022011--
011201001010001101020001011101100-0-110000001101-
301010021011001120111100110020001000100010000100103110000000101001000011211-
0000000000001101010011011011100020110001011100102110000101100000011
      Sciades_leptaspis             120-00000111130000001011-0-1-
002201010011201001010001101000001001101100-0-110000001101-
3010100210110011201111001100100010001000100001001010100000001010010000112-1-
0000000000001101010011011011100020110001011100102110000101000000011
      Occidentarius_platypogon      110-00000111130000001011110000112011--
011001011010001101020001001101100-00110000001100-
301010001010001020111100110010001000100010000100101010000000111001000011211-
000000000000110101001101101101110011000101110010211000010100000000?1
      Sciades_parkeri               -20-00000100130000001011-0-1-0112011--
011201001010001101020001111101100-0-110000001101-
301010021011001120111100110020004000100010000100103100000000101001000011201000000
000000001101010011011011100020110001011110202110000101000001011
      Sciades_passany               120-00000100130000001011-0-1-0022011--
0112010010100011010?0001111101100-0-110000001101-
3010100210110011201111001100200040000000100001001031000000?0101001000011201000000
000000001101010011011011100020110001011100102110?00101100000011
      Sciades_proops                -20-00000100130000001011-0-1-0112011--
011201101010001101020001111101100-0-110000001101-
301000021011001120111100110020004000000010000100103100000000101001000011201000000
000000001101010011011011100020110001011112202110000101000001011
      Sciades_sagor                 110-00000111130000001011-0-1-0112011--
011201001010001101020001001101100-0-11000000110--
301010021011001120111100110010001000100010000100101010000000101001000011201000000
000000001101010011011011100020110001011100102110000101000000011
      Sciades_seemanni              110-00000111130000001011-0-1-0112011--
011201001010001101020001001101100-0-110000001101-
301010021011001120111100110010001000100010000100101010000000101001000011211-
000000000000110101001101101110002011000?011100102110000101000000011
      Carlarius_gambensis           120-
0000011103001000101111100010201010111201001010100--
102000100110210100011000001110013010100210100010201111001100110010001000100000100
100000000000101001000011201000000000000001101010011011011100020110001011100102110
000101000000011
      Carlarius_heudelotii          120-
0000011103001000101111100010201010111201001010100--
102000100110210100011000001110013010100010100010201111001100110010101000100000100
100000000000101001000011201000000000000001101010011011011100020110001011100102110
000101000000011
      Carlarius_parkii              120-
0000011103001000101111100010201010111201001010100--
102000100110210100011000001110013010100210100010201111001100100010001000100000100
100000000000101001000011201000000000000001101010011011011100020110001011100102110
000101000000011
      Plicofollis_dussumieri        110-
00001112130000001011110000102000101112010021110111210411013111021110001100000110
```

```
10130101100101001102011110011001000110111022001010010001000010010100000300114 10-
000000100011111101011111110111000201100010111001032101001010000000111
      Plicofollis_nella           110-
0000111213000000101111000010200010111201002111011121041101311102111000110000011 0
10130101100101001102011110011001000110111022001010010001000010010100000300114 10-
000000100011111101011111110111000201100010111001032101001010000000111
      Plicofollis_platystomus     110-
0000010113000000101111100010200010011101001110000--
1041101011102111000110100011010130101000101001102011110011001000101000002001011 0
1000100001001010010300114010000000000000011010100111110110000101100000111001021 10
10010100000111
      Plicofollis_polystaphilodon  110-
0000111213000000101111000010200010111201002111011121041101311102111000110000011 0
10130101100101001102011110011001000110111022001010010001000010010100000300114 10-
000000100011111101011111110111000201100010111001032101001010000000111
      Plicofollis_tenuispinis     110-
0000010013000000101111000010200010011101001110001121041101311102111000110000011 0
1003010110010100110201111001100100011011102200101001000100001011010000300114010 0
00000100010111101011111110110000101100010111001032101001010000000111
      Potamosilurus_macrorhynchus  -10-
000001010300100010110110001020101001120100111000110-----
0110110211100011000001110013010100210100010201111001100100010101000100101101000 1
0000000101001000011201000000001000000101010011111011100020110000?11001021100001 0
1000000011
      Potamosilurus_latirostris    110-0000011103001000100101 2011--
2010100112010010100011010000011011021110001101000111001301010021010001020111100 1
1001000101010001001011010001000000010100100001020100000000100000010101001111011 1
000201100000111001021100001010000000011
      Amphiarius_phrygiatus       110-000001010321200010010 12011--
201010011001011110100--1050001111102111120110110020 03--
301010021010001020111100110010001010100010000110101010000000010100100001121 1-
000000000000110101001101101101110111000010110010111010010100000000 1
      Amphiarius_rugispinis       110-000001010321200010010 12011--
201010111001011110100--1050001111102111120110110020 03--
301010021010001020111100110010001010100010000110101010000000010100100001121 1-
000000000000110101001101101101110111000010110010111010010100000000 1
      Cryptarius_truncatus        010-
0000010103112000100001100010201000011011003010100--1030000-
0110211112011011002101003010100210100010201110001100100020101020210201001010110 1
0-0010100100001001 1-
0100000000001101010111011011010100110000101100103110110101000000 001
      Qarmoutus_hitanensis        110?0000010013000???011-0-1-0001001--
0????101101000100?????0??011101010000--
00000??????????????????????????10?????????200?????001--
10010????????????????????????????????????????????????????????1?1???100????0100??
?201001??????????-

;
END;
ctype ord: 2 12 14-15 17 27 32 35 42 55 65 68 73 82 88 96 103-104 106-108 116
126 131 146 150 177 187-188 190 196 204 212-214;
```
